# Genome-Wide Analysis of Cation/Proton Antiporter Family in Soybean (*Glycine max*) and Functional Analysis of GmCHX20a on Salt Response

**DOI:** 10.3390/ijms242316560

**Published:** 2023-11-21

**Authors:** Qi Jia, Junliang Song, Chengwen Zheng, Jiahui Fu, Bin Qin, Yongqiang Zhang, Zhongjuan Liu, Kunzhi Jia, Kangjing Liang, Wenxiong Lin, Kai Fan

**Affiliations:** 1 Key Laboratory for Genetics Breeding and Multiple Utilization of Crops, Ministry of Education/College of Agriculture, Fujian Agriculture and Forestry University, Fuzhou 350002, China; xgrz@163.com (J.S.); m13299716610@163.com (C.Z.); 1220102003@fafu.edu.cn (J.F.); 15776583358@163.com (B.Q.); liangkj_2005@126.com (K.L.); 2 Key Laboratory of Crop Ecology and Molecular Physiology, Fujian Agriculture and Forestry University, Fujian Province University, Fuzhou 350002, China; lwx@fafu.edu.cn; 3 Fujian Provincial Key Laboratory of Agroecological Processing and Safety Monitoring, College of Life Sciences, Fujian Agriculture and Forestry University, Fuzhou 350002, China; yqzhang@fafu.edu.cn (Y.Z.); liuzj@fafu.edu.cn (Z.L.); kjia@fafu.edu.cn (K.J.)

**Keywords:** cation/proton antiporter, soybean, *GmCHX20a*, salt response

## Abstract

Monovalent cation proton antiporters (CPAs) play crucial roles in ion and pH homeostasis, which is essential for plant development and environmental adaptation, including salt tolerance. Here, 68 CPA genes were identified in soybean, phylogenetically dividing into 11 Na^+^/H^+^ exchangers (NHXs), 12 K^+^ efflux antiporters (KEAs), and 45 cation/H^+^ exchangers (CHXs). The *GmCPA* genes are unevenly distributed across the 20 chromosomes and might expand largely due to segmental duplication in soybean. The GmCPA family underwent purifying selection rather than neutral or positive selections. The *cis*-element analysis and the publicly available transcriptome data indicated that GmCPAs are involved in development and various environmental adaptations, especially for salt tolerance. Based on the RNA-seq data, twelve of the chosen *GmCPA* genes were confirmed for their differentially expression under salt or osmotic stresses using qRT-PCR. Among them, *GmCHX20a* was selected due to its high induction under salt stress for the exploration of its biological function on salt responses by ectopic expressing in *Arabidopsis*. The results suggest that the overexpression of *GmCHX20a* increases the sensitivity to salt stress by altering the redox system. Overall, this study provides comprehensive insights into the CPA family in soybean and has the potential to supply new candidate genes to develop salt-tolerant soybean varieties.

## 1. Introduction

Plants have orchestrated complex systems to maintain ion and pH homeostasis, which is critical for environmental adaptation and development. Previous evidence has shown that transmembrane ion channels and transporters are crucial in the regulation of ion and pH balance [1,2]. One of the important membrane transporter families is the monovalent cation/proton antiporter (CPA) family, which can exchange monovalent cations, mainly Na^+^, K^+^, and H^+^, across the membrane [3]. CPA members have been identified to broadly exist in bacteria, fungi, plants, and metazoa [4,5].

In plants, the CPA family is usually grouped into two subfamilies, CPA1 (2.A.36) and CPA2 (2.A.37), with all members containing the conserved Na^+^/H^+^ exchanger (PF00999) domain [6]. The CPA1 subfamily includes the intracellular Na^+^/H^+^ exchangers (NHXs) and the plasma membrane-bound NHXs (NHX7/SOS1(overly salt-sensitive 1) and NHX8), which contain 10–12 membrane-spanning domains. On the other hand, the CPA2 subfamily is composed of the K^+^ efflux antiporters (KEAs) and the cation/H^+^ exchangers (CHXs), which have 8–14 membrane-spanning domains [5]. In spite of their essential function in plant growth and development, they have been identified to play crucial roles in abiotic stress tolerance, especially in salt responses [7,8]. The best-characterized CPA members with key roles in salt tolerance usually belong to the NHX-type cation/H^+^ antiporters, such as AtNHX1 and AtSOS1 [9]. AtNHX1 and its homolog AtNHX2 have been shown to be major contributors in the vacuolar pH regulation and sequestration of K^+^ and Na^+^ [10]. AtSOS1, one of the important components in the SOS pathway, has been proved to play crucial roles in Na^+^ extrusion under salt stress [11,12]. Evidence has showed that KEAs can function in K^+^ homeostasis and osmotic adjustment in *Arabidopsis* [13,14,15]. The CHX subfamily is the biggest CPA subfamily, but only a few CHXs have been understood for their biological function and molecular mechanisms [7].

Except for the model plants *Arabidopsis* and rice, CPA family members have been systematically explored at the whole genome level in several plant species, such as grapevine, pear, radish, bread wheat, maize, and moso bamboo [16,17,18,19,20,21,22]. Due to their conserved structure for cation transportation, more attention has been paid to their potential function on salt and osmotic tolerance, especially for the NHX-type exchangers in crops [20,22,23,24,25]. Soybean (*Glycine max*) is an important legume crop supplying premium oil and protein, which is used in human diets, animal feeds, and biodiesel production worldwide. Soil salinity is a big threat for the productivity and the quality of soybean [26]. Identification of novel salt-responding genes provides promising clues to reveal the mechanism of salt tolerance and improve salt tolerance in soybean breeding.

Recent reports showed that CPA members play a crucial role in salt tolerance in soybean. *GmCHX1*/*GmSALT3/Ncl* has been proved to be the dominant gene underlying the major quantitative trait locus (QTL) for salt tolerance in soybean [27,28,29,30]. The results indicated that *GmCHX1*/*GmSALT3/Ncl* is involved in the root exclusion of both Na^+^ and Cl^−^, together in relation to K^+^ homeostasis under salt stress [29,31,32]. Evidence has also implied that *GmCHX1/GmSALT3/Ncl* facilitates reactive oxygen detoxification under salt stress [33,34]. According to this gene, efficient PCR-based markers have also been developed for the selection of salt-tolerant or -sensitive soybean germplasm [35]. Another CHX-type transporter gene *GmCHX20a*, which is adjacent to *GmCHX1* at the major salt-tolerant QTL, has been identified to direct Na^+^ flux in an opposite direction to *GmCHX1*, suggesting that GmCHX20a and *GmCHX1* might work complementally to tolerate both osmotic stress and ionic stress from salinity [36]. Additionally, GsCHX19.3 from wild soybean (*Glycine soja*) has been characterized for its function in salt–alkaline tolerance by mediating K^+^ uptake [37]. Considering the crucial roles of the NHX-type exchangers on salt tolerance, several trials have been performed in soybean as well, especially for *GmNHX1* and *GmSOS1* [25,38,39,40,41,42,43,44,45]. All these reports indicated that *GmSOS1*, *GmNHX1*, *GmNHX2*, *GmNHX5*, and *GmNHX6* might play important roles in salt tolerance, mostly working by maintaining Na^+^ and K^+^ homeostasis. Due to their potential significant function, several investigations have been made at the genome-wide level, but only for part of the soybean CPA family [23,25,46]. However, a systematic exploration of the whole CPA family is lacking. There is some ambiguity for the CPA nomenclature. *GmNHX1* is represented for different genes in different reports. *GmCHX1* was named as the first CHX-type exchanger characterized from soybean without considering its homology. This study aims to conduct a genome-wide identification of the *GmCPA* genes and perform evolutionary and expression analyses of the retrieved *GmCPA* genes. Moreover, *GmCHX20a* was further verified for its function in salt response.

## 2. Results

### 2.1. Identification and Classification of CPA Genes in Soybean

In order to identify the CPA proteins in soybean, an HMM search and a BLASTP search were performed against the soybean proteome database G. max Wm82.a2.v1, with seed sequences of PF00999 downloaded from the Pfam database. The two results were merged to obtain 134 sequences of peptides. Subsequently, each retrieved sequence was confirmed with the presence of PF00999 using the InterPro database. Those that did not contain the PF00999 domain were removed. In addition, two short sequences of Glyma.15G200200.1 (117 amino acids) and GlymaU041300.1 (169 amino acids) were removed due to their incomplete PF00999 domain. A total of 95 transcripts encoded by 68 genes were identified (Table S1).

According to previous studies on the plant CPA family, the CPA family is often divided into three subfamilies, which are NHX, KEA, and CHX [5,6,18]. In this study, the soybean CPA genes were designated based on their homology with *Arabidopsis* genes using a similar rule with modifications [37]. If more than one soybean gene was homologous to one *Arabidopsis* CPA gene, the alphabetic character was added to distinguish them, such as *GmNHX5a*, *GmNHX5b*, and *GmNHX5c*. Moreover, the following numbers indicated alternative transcripts of the same gene, for example, *GmNHX5c.1*, *GmNHX5c.2*, *GmNHX5c.3*, and *GmNHX5c.4* [47]. Compared to soybean NHX (16.7%) and CHX (11.1%) genes, a larger proportion (66.7%) of soybean KEA genes were predicted to contain alternative transcripts, suggesting soybean KEA genes regulate their function via proteomic diversity or differential gene expression [48]. In addition, the previously reported salt-determinant CHX gene, *GmCHX1*/*GmSALT3* [27,28,30], is renamed as *GmCHX20b* here due to its high similarity to AtCHX20. The full-length form of *GmCHX20b* only exists in the salt-tolerant accessions. In the salt-sensitive accessions, such as Williams 82 and C08, the gene is broken into two parts, *GmCHX20b1* and *GmCHX20b2*, using a Ty1/copia retrotransposon [27].

**Figure 1:**
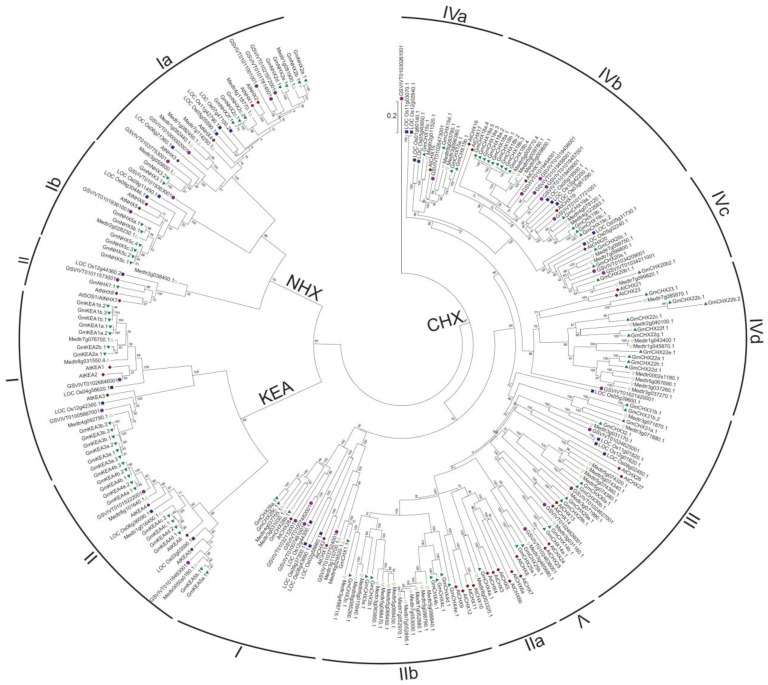
Phylogenetic analysis of the CPA proteins in *Glycine max* (*Gm*), *Arabidopsis thaliana* (*At*), *Oryza sativa* (*Os*), *Vitis vinifera* (*Vv*), and *Medicago truncatula* (*Medtr*). An unrooted neighbor-joining tree was generated based on the alignment of full-length protein sequences of CPAs using the *p*-distance method and a bootstrap value of 1000. The CPAs are classified into three subfamilies: NHX, KEA, and CHX. The arcs indicate the subgroups of the CHX subfamily. The scale bar represents 0.2 amino acid substitutions per site. The different color labels for the putative proteins indicate that the CPA proteins are from different plants. Green solid triangle stands for soybean. Red solid diamond stands for *Arabidopsis*. Blue solid square stands for rice. Purple solid circle stands for grape. The triangle stands for *Medicago truncatula*.

To classify soybean CPAs, a neighbor-joining phylogenetic tree was constructed using the full-length sequences of CPA proteins from *Glycine max* (*Gm*), *Arabidopsis thaliana* (*At*), *Oryza sativa* (*Os*), *Vitis vinifera* (*Vv*), and *Medicago truncatula* (*Medtr*) (Figure 1). Based on the phylogenetic analyses, the soybean CPA proteins (GmCPAs) were classified into three main subfamilies: NHX (16 putative proteins encoded by 11 genes), KEA (24 putative proteins encoded by 12 genes), and CHX (55 putative proteins encoded by 45 genes). As expected, the CPAs from rice, the monocot plant, always separate from those from the dicot plants, *Arabidopsis*, soybean, and *Medicago truncatula*. The genes from legume are more likely to cluster together. The NHX and KEA subfamilies could be further divided into three and two subgroups, respectively, which is consistent with previous studies [5,6,23,49]. Relatively, the plant CHX subfamilies always exhibit more diversity with more members than the NHX and KEA subfamilies. H. Sze et al. have classified the *Arabidopsis CHX* genes into five groups [49], and S. Chanroj et al. have divided the *CHX* genes from 15 plant species into eight subgroups [5]. Based on those, we separated the *CHX* genes into nine subgroups (Figure 1). Notably, the subclade containing *GmCHX31a* and *GmCHX31b* in the subgroup CHXIII contains only legume genes, suggesting that it is legume-specific. In the subgroup CHXIII, there are no *Arabidopsis* homologous genes in the two subclades, one containing *GmCHX32* and the other containing *GmCHX29a*, *GmCHX29b*, *GmCHX30a*, and *GmCHX30b*. However, there is one grape homologous gene in each of those two subclades, suggesting that those genes are disappeared in *Arabidopsis* and remain in the legume during the evolution. The subgroups of CHXIIa, CHXIIb, CHXIVd, and CHXV are eudicot-specific, and there are also legume-specific subclades within them. This indicates that these genes diverge later in the specification of the legume. The multiple genes in those subgroups could probably be a result of additional duplications [5,50].

The basic characteristics of GmCPAs are shown in Table S1, including the number of introns, molecular weight, isoelectric point (pI), number of transmembrane helixes, and predicted subcellular localization. The molecular weights (KDa) of the GmCPA proteins range from 39.65 (*GmCHX22b.2*) to 129.82 KDa (*GmKEA1b.1* and *GmKEA1b.3*) and the predicted pI from 4.9 (*GmKEA1b.2*) to 9.75 (*GmCHX20b.1*). Most of the GmCPA proteins are predicted to contain 10–12 transmembrane helixes and locate at the plasma membrane or intracellular membrane system.

### 2.2. Gene Structure and Conserved Domain Analysis

**Figure 2:**
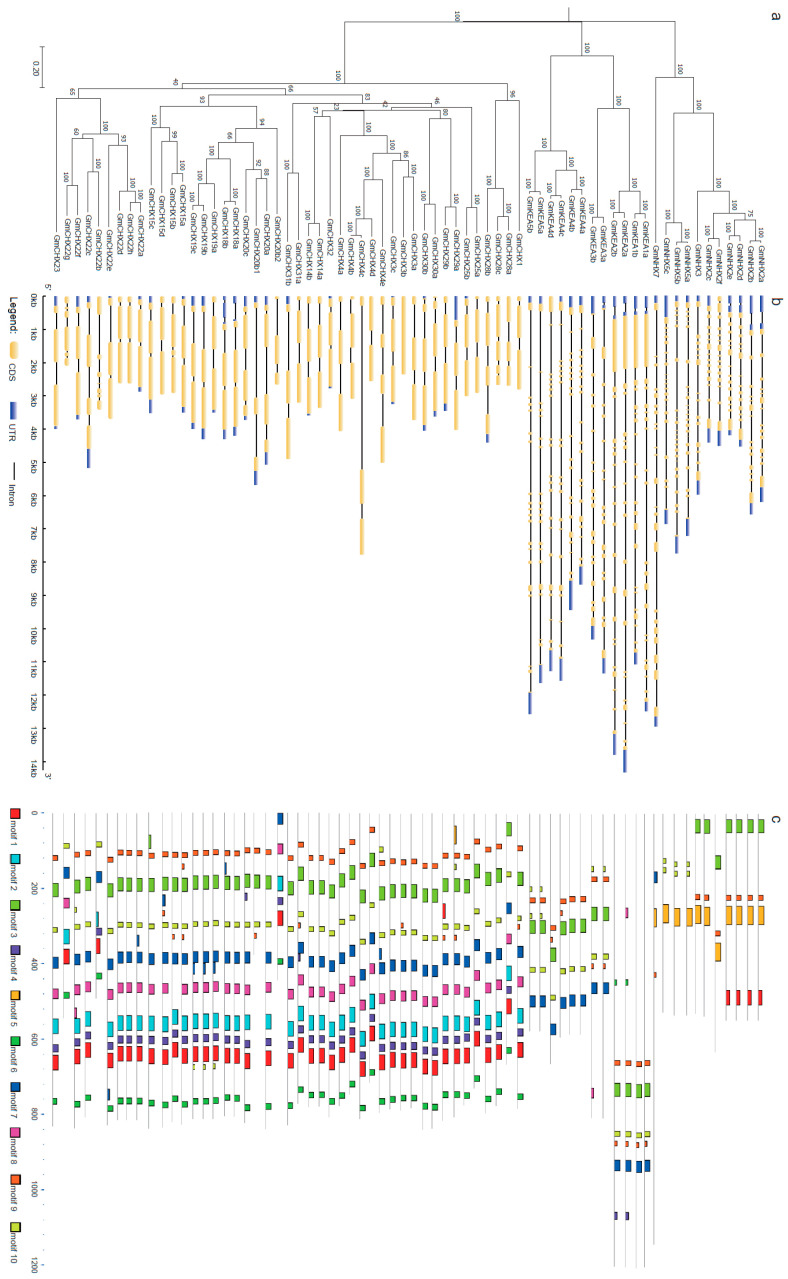
Gene structures and conserved motifs of the soybean CPA family. (**a**) Phylogenetic relationships of the *GmCPA* family. The unrooted neighbor-joining tree was constructed with a bootstrap value of 1000 using Mega11. The scale bar represents 0.2 amino acid substitutions per site. (**b**) The exon–intron structures of the *GmCPA* genes. The gene structures were created by the GSDS_2.0_ program. Exons, introns, and untranslated regions (UTRs) are indicated by yellow rectangles, thin lines, and blue rectangles, respectively. The scale represents the length of DNA sequence. (**c**) The conserved motifs of *GmCPA* proteins. The ten motifs were identified by MEME and are represented by the colored boxes. Each motif is indicated by a specific color. The information of the conserved motifs is shown in Table S2.

Gene structural diversity may contribute to the evolution process of multi-gene families [47,51]. Thus, the exon–intron organization of soybean *CPA* genes was analyzed using the GDSA online suite and compared to their phylogenetic relationships made by Mega6 with the neighbor-joining method and a bootstrap value of 1000 (Figure 2). As expected, the genes of the same subfamily shared a similar gene structure, suggesting their close phylogenic relationship. The number of introns ranged from 0 to 22 and varied considerably among subfamilies (Table S1). The NHX subfamily contained 12–22 introns and KEA had 16–20 introns, whereas *CHX* contained only 0–6 introns. The length of the introns was greatly diverse, even in the same clade. This indicated that exon–intron loss and gain occurred during the evolution of the *GmCPA* family.

To further understand the divergence of *GmCPA* proteins, a total of 10 conserved motifs were identified in *GmCPA* by the MEME web server (Figure 2c and Table S2). Again, the members of the same phylogenic clade shared similar motifs, supporting our phylogenic construction of *GmCPA*. Most *GmCPA* members contain Motifs 3 and 9, which are fragments of the sodium/hydrogen exchanger family domain (PF00999). The sequence of Motif 3 is legume-specific. The other two motifs, Motifs 7 and 10, are also fragments of PF00999, which existed in the most *GmCHX* and *GmKEA* members, but not in GmNHXs. Motif 5, a fragment of PF00999, is specific in *GmNHXs*. In addition, Motifs 2, 4, 6, and 8 are specific for *GmCHXs*. Motif 1 only existed in some vacuolar localized *GmNHXs* and all the *GmCHXs*. This indicates the functional divergence among the different *GmCPA* subfamilies.

### 2.3. Chromosomal Locations and Expansion Pattern of GmCPAs

**Figure 3:**
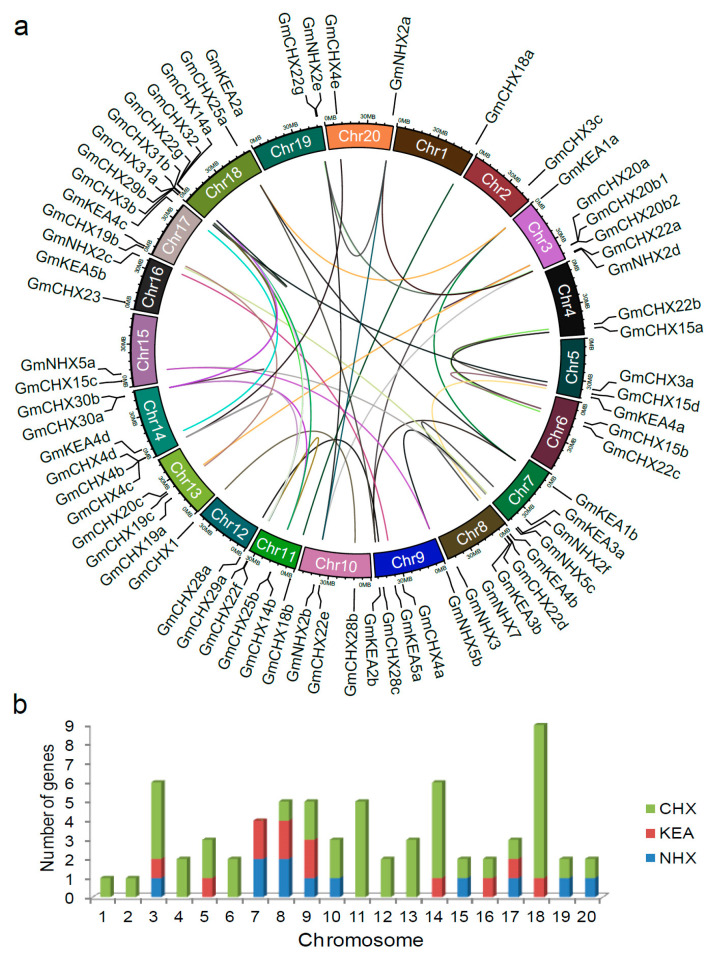
Chromosomal distribution of the *CPA* genes in soybean. (**a**) Schematic of the localization and inter-chromosomal relationships of *GmCPA*s. The *GmCPA* genes were mapped on the soybean chromosomes. The duplicated gene pairs were linked by the colored lines. (**b**) Chromosomal distributions of the three CPA subfamilies in soybean.

Duplication events have been recognized as the main cause for the expansion and functional diversification of a gene family in evolution [52]. To determine their evolutionary relationships, their locations were mapped to the soybean chromosomes, and the duplicated gene pairs were plotted against the chromosomal position (Figure 3a and Figure S1). The 68 *GmCPA* genes are unevenly distributed on all the soybean chromosomes, with chromosome 18 containing the largest number of genes (9) and chromosome 1 harboring the fewest (1) (Figure 3b). Chromosomes 3, 8, 9, and 17 possess all three types of the *GmCPA* genes, whereas the other chromosomes harbor one or two types.

During the process of evolution, two more rounds of whole-genome duplication events, legume-common tetraploidization (~60 Mya), and soybean-specific tetraploidization (~13 Mya) occurred in the soybean genome compared to those in grape (*Vitis vinifera*), which is often taken as the genome structural reference for eudicots [50,53]. The enormous size of the *GmCPA* gene family indicated that it has evolved through duplications during those polyploidization events. However, the numbers of the three CPA subfamily genes are less in soybean than expected, suggesting that gene loss events might happen. Due to the soybean-specific tetraploidization, the number of homolog genes was doubled in soybean compared to *Medicago truncatula*. There are 47 *CHX* genes in *Medicago truncatula* and 45 in soybean, indicating that some of the *CHX* genes might be lost in the soybean-specific tetraploidization.

Based on the comparison with the plant genome duplication database (PGDD) and BLASTP, we found that 55 of the 68 identified genes (81%) were duplicated, with a total of 49 paralogous gene pairs in the *GmCPA* gene family (Table S3). Among them, 43 pairs result from segmental duplications and 6 pairs from tandem duplications, suggesting that segmental duplication might be a major driving force for the expansion of *GmCPA*s. The divergence dates range from 0.77 Mya to 174.84 Mya. The Ka/Ks ratios of the duplicated *GmCPA* gene pairs varied from 0.06 to 1.19. The Ka/Ks value of most pairs are less than 1, and only four pairs exhibit a value of more than 1, indicating that they had undergone a strong purifying selection.

### 2.4. cis-Elements Analysis in the Promoter of GmCPAs 

In order to explore the potential biological functions for *GmCPAs*, the promoter region (2000 bp upstream from the start codon) of each *GmCPA* gene was investigated for *cis*-elements analysis via PlantCARE. The predicted results showed that a total of 102 types of *cis*-elements were identified (Table S4), and all the *GmCPA* genes contained core promoter elements, such as TATA box and CAAT box, in the promoter regions. Based on the functional annotations of those predicted *cis*-elements, they were divided into light-responsive, phytohormone responsive, stress-responsive, and development-related elements. 

**Figure 4:**
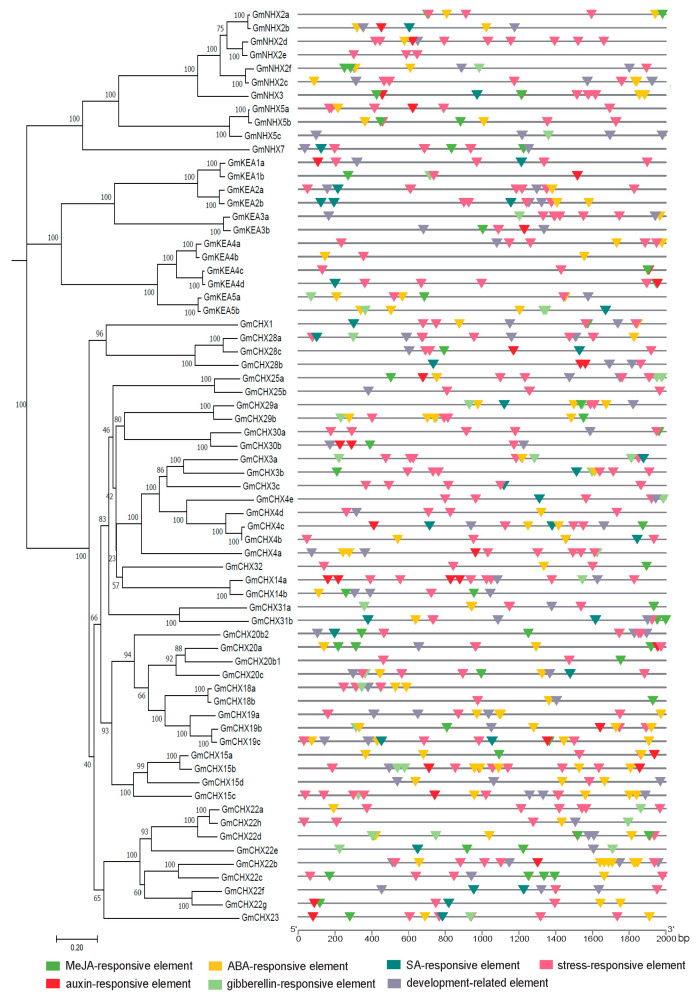
Distribution of *cis*-elements in the promoter regions of the *GmCPA* genes. The 2 kb sequences upstream of the identified *GmCPA* genes were submitted to PlantCARE for predicting *cis*-elements. The left panel is the phylogenetic clustering of *GmCPAs*, and the right pane is the pattern of the predicted *cis*-elements, which are present by the lines of distinct colors. A scale of promote length is indicated at the bottom.

The distribution and number of functional annotations are indicated in Figure 4 and Table S5. Interestingly, the light-responsive elements were again present in all the *GmCPA* promoters, which have also been reported in other plant *CPA* genes, such as maize [20] and *Phyllostachys edulis* [21]. The identified hormone-responsive elements existed broadly in *GmCPA*s, including methyl jasmonate (MeJA)-, abscisic acid (ABA)-, salicylic acid (SA)-, auxin (IAA)- and gibberellin (GA)-responsive elements. Among them, ABA- (48 of the 68 *GmCPA* genes) and MeJA-responsive (38 of 68 *GmCPA*s) elements appeared more frequently. Most *GmCPA*s contained 2–3 hormone-responsive elements in their promoter regions. *GmCHX23* harbored the *cis*-elements involved in all the above five hormone-responsive elements. Among the 68 *GmCPA* genes, 62 of them contained stress-responsive elements, including TC-rich repeats (defense and stress responsiveness) in 29 *GmCPA*s, MBS (MYB binding site involved in drought inducibility) in 35 *GmCPA*s, LTR (low-temperature responsiveness) in 18 *GmCPA*s, WUN-motif (wound responsive) in 7 *GmCPA*s, and two anaerobic induction elements (ARE and GC-motif) in 53 *GmCPA*s. In the aspect of the development-related elements, various types were identified and distributed separately. Overall, our results suggested that the *GmCPA* genes play potential roles in light-, hormone-, and stress-responses and development.

### 2.5. Expression Analysis of GmCPAs in Different Tissues

**Figure 5:**
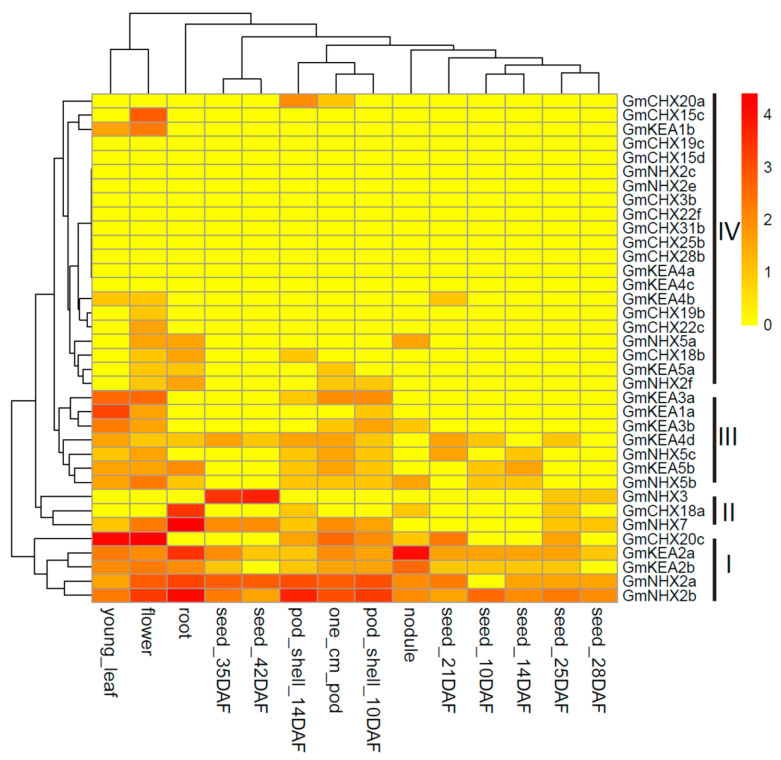
Tissue-specific expression profiles of the *GmCPA* genes. The transcriptomic data retrieved from Soybase were analyzed for 14 tissues in soybean. The heatmap was constructed by R software version 3.6.2 with the “pheatmap” package. According to hierarchical clustering, *GmCPA*s were divided into 4 groups (I–IV). The color scale on the right indicates the gene expression level. DAF: days after flowering. cm: centimeter.

Tissue-specific expression is likely related to biological function for each gene. To investigate the expression profiles of the *GmCPA* genes in different tissues, the publicly available RNA-seq atlas data were extracted from Soybase for 14 different tissues in the soybean cultivar Williams 82, including young leaf, flower, 1 cm pod, pod shells at different stages, seeds at different stages, root, and nodule [54,55]. The transcriptomic data were obtained for 64 of the 68 *GmCPA* genes, except for *GmCHX4c*, *GmCHX20b1*, *GmCHX20b2*, and *GmCHX22b*, suggesting that these four genes are pseudogenes or only expressed at specific developmental stages or under particular conditions. Among the detected 64 *GmCPA* genes, 36 *GmCPA* genes expressed at least one of the fourteen tissue types, which were used for cluster analysis (Figure 5). Most *GmCPA* genes represented distinct expression patterns. As revealed by the heatmap, *GmCPA*s were divided into four groups (I–IV). The *GmCPA* genes in Group I displayed higher expression levels in most of the studied tissues than the other genes, suggesting their constitutive roles during plant development. The *GmCPA* genes in Group II showed specific expressions in root or seed. Meanwhile, the *GmCPA* genes in Group III exhibited a medium expression level throughout the entire plant, and most of the Group IV genes had the lowest expression level compared with the other groups. Particularly, *GmCHX15c* had a high expression only in flower, *GmNHX3* only in seed, and *GmCHX20a* only in pod, demonstrating its specific function in the respective tissue. Notably, the homologous genes, *GmKEA2a/2b* and *GmNHX2a/2b*, shared similar expression patterns, suggesting their redundant roles in development.

### 2.6. Expression Profiles of GmCPAs under Salt Stress and Osmotic Stress 

**Figure 6:**
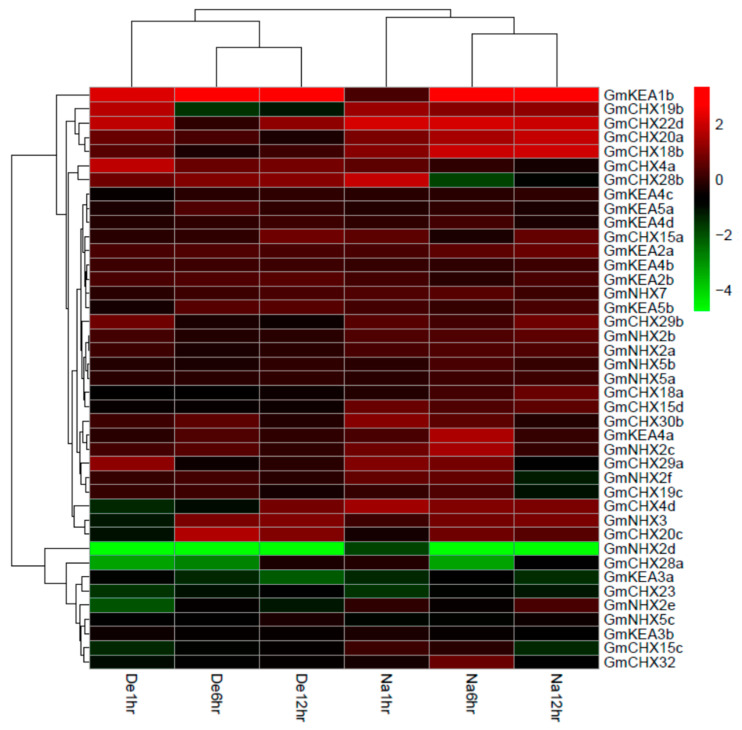
Expression patterns of the *GmCPA* genes in response to salt or dehydration stress. The transcriptomic data (GSE5252) obtained from the soybean roots were downloaded from the NCBI GEO database. A heat map with clustering was created based on the log2 conversion values of the relative expression data compared with the controls. The right color scale represents the expression level, with green for the reduced expression and red for the up-regulation expression. De1hr, De6hr, and De12hr indicate the dehydration treatments for 1 h, 6 h, and 12 h, and Na1hr, Na6hr, and Na12hr salt treatments for 1 h, 6 h, and 12 h, respectively.

Previous reports have shown that the members of the CPA family could play an important role in plant salt tolerance, such as NHX1, NHX2, SOS1, and so on [1,7]. Some soybean CPA members have been identified to perform critical function in salt responses as well, for example, *GmCHX1*/*GmSALT3* (renamed as *GmCHX20b* in this study), *GmCHX20a*, *GsCHX19.3* (the closest homolog in *Glycine max* is *GmCHX19b*), *GmNHX6* (renamed as *GmNHX5b* here), and *GmNHX5* (renamed as *GmNHX5a* here), as well as the soybean homologs of NHX1, NHX2, and SOS1 [25,27,28,30,31,32,33,34,36,37,42,44]. In addition, it is believed that the early threat of salt stress to plants is osmotic harm [56]. The publicly available RNA-seq data (GSE5252) were analyzed here to explore the potential function of plants under salt stress or dehydration stress, which is highly similar to drought or osmotic stress [57]. The soybean transcriptomic data of 41 *GmCPA* genes under salt or dehydration stress were retrieved to make the heat map (Figure 6). The differentially expressed genes were considered by a 2- or more fold change in the expression level under salt or dehydration treatments of at least one time point with an FDR (false discovery rate) less than 0.05. A total of 22 *GmCPA*s were selected, out of which there were only 7 under salt stress, 4 under dehydration stress, and 11 for both, indicating that *GmCPA*s are involved in salt and dehydration responses, probably playing more crucial roles in the responses to salt stress than dehydration. Meanwhile, more transcriptomic data from cultivated soybean (C08) and wild soybean (W05) under salt treatments were utilized to further explore the potential function of *GmCPAs* in salt responses [58]. Based on this study, the transcriptomic data of 32 *GmCPA* genes were retrieved as shown in the heat map (Figure S2). With the above criteria, 26 *GmCPA*s were considered as the deferentially expressed genes.

**Figure 7:**
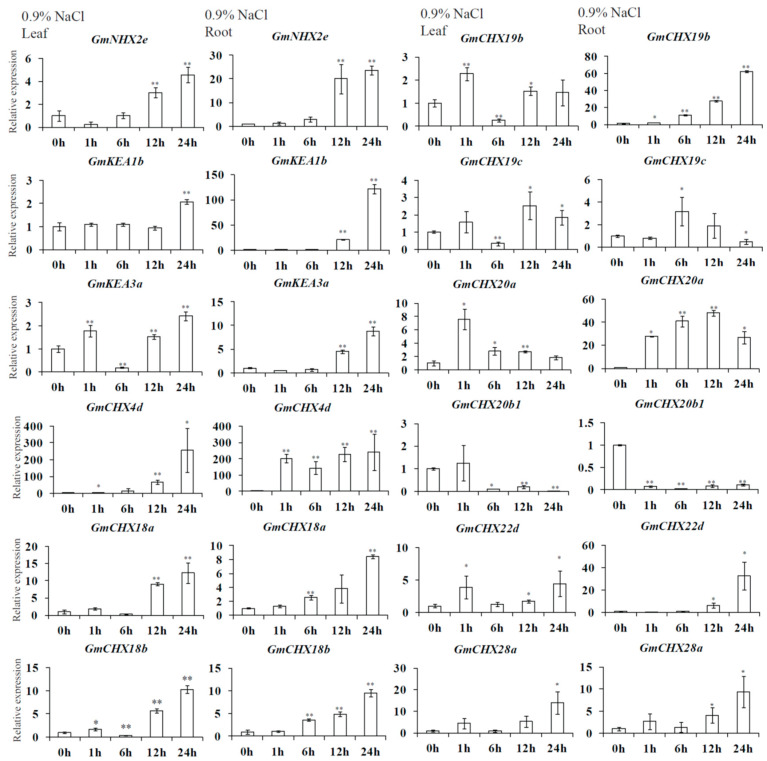
Expression levels of 12 *GmCPA*s in leaves and roots under salt stress using qRT-PCR. The soybean plants were treated with 0.9% NaCl for at 0 h, 1 h, 6 h, 12 h, and 24 h. *GmELF1b* was taken as the reference gene. The relative gene expression levels were calculated relative to the data at 0 h using the 2^−ΔΔCt^ method. Mean values and standard deviations (SDs) were obtained from three biological and three technical replicates (*t*-test, * *p* < 0.05, ** *p* < 0.01).

**Figure 8:**
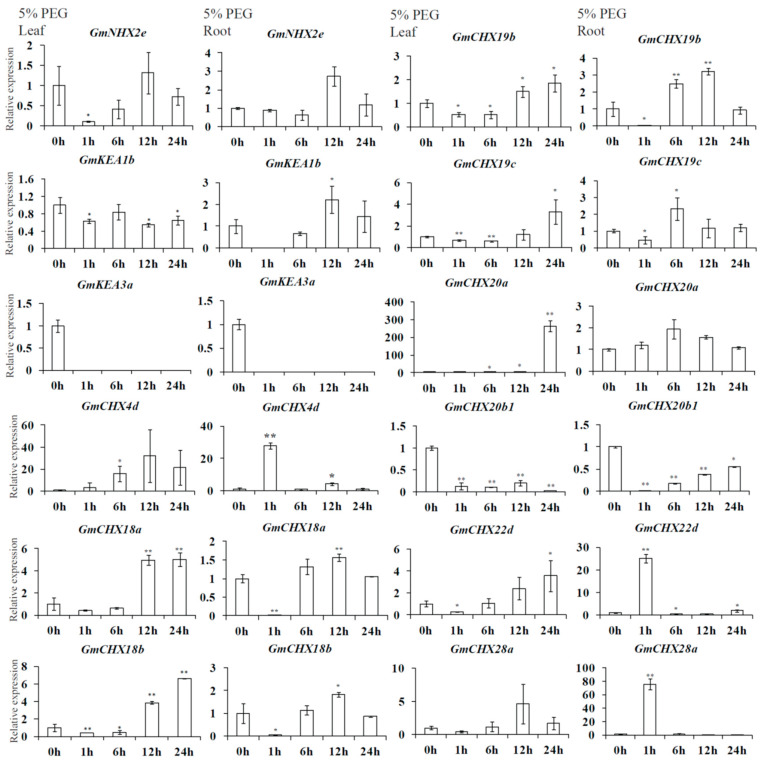
Expression levels of 12 *GmCPA*s in leaves and roots under osmotic stress using qRT-PCR. The soybean plants were treated with 5% PEG for at 0 h, 1 h, 6 h, 12 h, and 24 h. *GmELF1b* was taken as the reference gene. The relative gene expression levels were calculated relative to the data at 0 h using the 2^−ΔΔCt^ method. Mean values and standard deviations (SDs) were obtained from three biological and three technical replicates (*t*-test, * *p* < 0.05, ** *p* < 0.01).

Combining the two transcriptomic data, 13 candidate *GmCPA* genes were selected for differential expression under salt or dehydration stresses, including *GmNHX2e, GmKEA1b, GmKEA3a, GmCHX4d, GmCHX18a, GmCHX18b, GmCHX19b, GmCHX19c, GmCHX20a, GmCHX20b1, GmCHX22d, GmCHX28a,* and *GmCHX28b*. Their expression levels were verified in leaves and roots under salt or osmotic stress by quantitative reverse-transcription PCR (qRT-PCR) (Figure 7 and Figure 8), except no signals were detected for *GmCHX28b*. The roots and leaves were collected separately from the plants treated by 0.9% NaCl or 5% PEG for different lengths of time (0 h, 1 h, 6 h, 12 h, and 24 h). In general, it seemed that the expression patterns detected using qRT-PCR were consistent with the RNA-seq data for the *GmCPA* genes. Among the detected genes, *GmCHX4d*, *GmCHX18a*, *GmCHX18b,* and *GmCHX20a* had induced expression levels in both leaves and roots under salt or osmotic stresses, suggesting that they might be involved in both stresses. *GmCHX4d* and *GmCHX20a* had impressive higher multiples of induced expression changes under salt or osmotic stresses. However, the relative expression levels of *GmCHX4d* were lower than that of *GmCHX20a*, indicating that *GmCHX20a* might play a more crucial role. On the other hand, the expression levels of *GmCHX20b1* were reduced in both the leaves and roots under salt or osmotic stresses. Meanwhile, the induced expression levels of *GmNHX2e*, *GmKEA1b*, and *GmCHX19b* were much higher under salt stress than osmotic stress, especially in the roots, suggesting that they are more related to salt stress than osmotic stress. The expressions of *GmCHX19c*, *GmCHX22d*, and *GmCHX28a* exhibited biphasic induction patterns in the roots and leaves. Unlike *GmCHX19c*, *GmCHX22d* and *GmCHX28a* have a contrary trend of expressional changes under salt stress to that under osmotic stress. This indicated that those genes play cooperative roles in salt and osmotic tolerance. Interestingly, only the expression of *GmKEA3a* had not been detected under osmotic stress, indicating that it is specifically suppressed by osmotic stress. In addition, it was highly induced at the late stage of salt stress, suggesting that it might be involved in the resistance of ionic harm.

### 2.7. Ectopic Expression of GmCHX20a Increased Salt Sensitivity in Arabidopsis 

**Figure 9:**
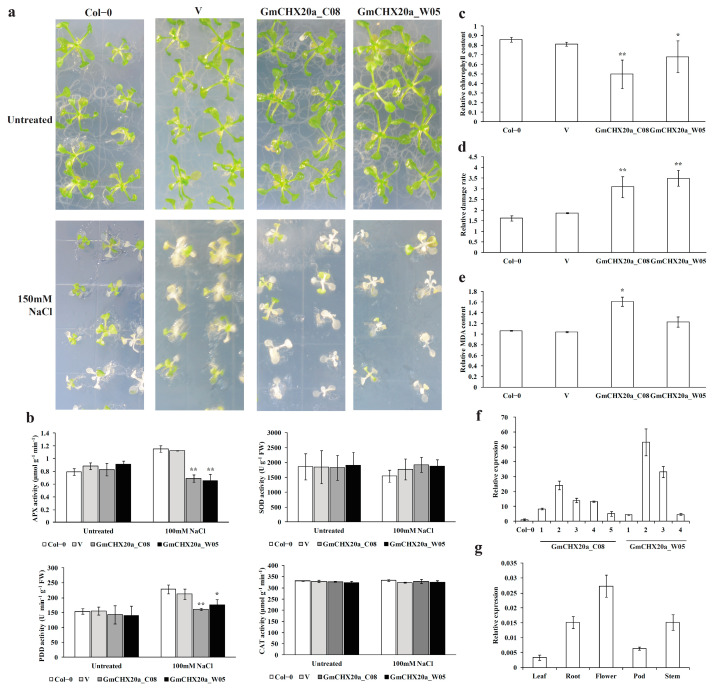
Functional analysis of GmCHX20a. (**a**) Phenotype of the *Arabidopsis* plants under salt treatments. The 7-day seedlings were transferred to the fresh 1/2 MS media with or without 150 mM NaCl for 14 days. (**b**) Activities of antioxidant enzymes in the transgenic *Arabidopsis* plants under salt stress. The 10-day seedlings were treated with or without 100 mM NaCl for 24 h. (**c**) Relative chlorophyll contents. (**d**) Relative damage rates. (**e**) Relative MDA contents. The 10-day *Arabidopsis* seedling were treated with or without 100 mM NaCl for 3 days to detect (**c**–**e**). (**f**) Expression analyses of *GmCHX20a* in the transformed *Arabidopsis* plants using qRT-PCR. (**g**) Expression analyses of *GmCHX20a* in different soybean tissues using qRT-PCR. Col-0 stands for the wild-type *Arabidopsis* plants, V for the control plants transformed with the empty vector, GmCHX20a_C08 for the *GmCHX20a_C08*-overexpressing plants, and GmCHX20a_W05 for the *GmCHX20a_W05*-overexpressing plants. The error bars indicated for standard deviations from three replicates. The data were analyzed using ANOVA (Dunnett’s). Asterisk indicates the significant difference (* *p* < 0.05, ** *p* < 0.01).

Based on the expression analyses of the soybean *CPA* genes, *GmCHX20a* was selected due to its high induction in both cultivated soybean C08 and wild soybean W05 under salt stress. The *GmCHX20a* genes from C08 to W05 were cloned and transformed in *Arabidopsis*, as previously described [36]. To further verify its biological function on salt tolerance, the phenotype and physiological indicators were detected in the transgenic plants under salt treatments. Both the *GmCHX20a*-transgenic plants (*GmCHX20a_C08* and *GmCHX20a_W05*) were similar as the wild-type (WT) plants and the control plants transformed with the empty vector (V) under a normal growth condition. When the one-week seedlings were treated with 150mM NaCl for 14 days, the transgenic plants suffered more damage than the controls (Figure 9a). Though there are minor difference for the *GmCHX20a_C08* genes and the *GmCHX20a_W05* genes [36], there seems to be no significant difference between their function on the salt responses.

Salt stress can cause the excessive accumulation of reactive oxygen species (ROS), which could be reduced by antioxidant enzymes, such as ascorbate peroxidase (APX), peroxidase (POD), superoxide dismutase (SOD), and catalase (CAT) [59]. Thus, the activities of the antioxidant enzymes were determined for the 10-day seedlings treated by 100 mM NaCl for 24 h (Figure 9b). Under normal growth conditions, the controls and the transgenic plants contained similar activities of the above antioxidant enzymes. When treated with salt stress, the activities of APX and POD were significantly reduced in the transgenic plants compared with the controls. There seemed to be no difference for the activities of SOD and CAT between the transgenic plants and the controls under salt stress. When treated with salt stress for more than 24 h, the H_2_O_2_ contents detected by the DAB staining again suggested that the *GmCHX20a*-transgenic plants generate more ROS (Figure S3), which is consistent with the results from the activities of antioxidant enzymes.

Meanwhile, the 10-day seedlings treated by 100 mM NaCl for 3 days were detected for stress-responding physiological indicators, including chlorophyll levels, electrical conductivity (EC), and malondialdehyde (MDA) contents. The relative chlorophyll contents of the transgenic plants were significantly lower than the WT and the empty vector-transformed control plants, suggesting that the photosynthetic capacity was inflected (Figure 9c). Electrical conductivity reflects electrolyte leakage, which was used to assess membrane permeability [60]. Based on the electrical conductivity, the relative damage rates were calculated from the ratio between the salt-treated plants and their respective controls under a normal growth condition. The results showed that the relative damage rates of the transgenic plants were significantly higher than the controls under salt stress (Figure 9d). The MDA content reflects the degree of lipid oxidative damage, which could lead to cell membrane damage [61]. The results showed that the relative MDA contents of the transgenic plants were higher than the controls under salt stress as well (Figure 9e). The *GmCHX20a* transgenic plants were verified using qRT-PCR, indicating that all of them contained the ectopic expression of *GmCHX20a* (Figure 9f).

**Figure 10:**
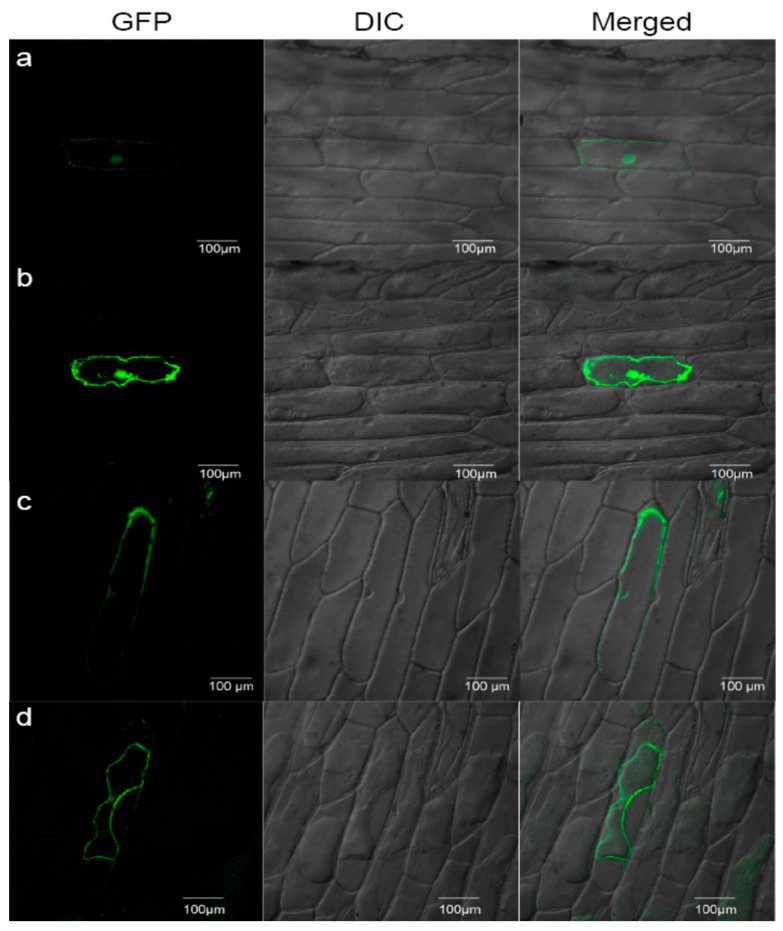
Subcellular localization of *GmCHX20a* in onion epidermal cells. Transient expression of free GFP (**a**,**b**) or the GmCHX20a-GFP fusion protein (**c**,**d**) was conducted in onion epidermal cells before (**a**,**c**) or after (**b**,**d**) plasmolysis. GFP fluorescence, bright-field, and merged images are shown. Scale bars indicate 100 μm.

In addition, the expression pattern of *GmCHX20a* in different tissues has also been detected in soybean (Figure 9g). The expression of *GmCHX20a* was detected in all the soybean tissues. Flowers, roots, and stems contained a higher expression level than leaves and pods. In addition, *GmCHX20a* was fused with *GFP* under the control of the 35S promoter in onion epidermal cells to determine its subcellular localization (Figure 10). The GFP protein appeared throughout the entire cell, whereas the *GmCHX20-GFP* fusion protein was observed in the plasma membrane, indicating that it plays a role in ion exchange with theoutside. Combining the expression analyses of soybean under salt stress, it was implied that *GmCHX20a* performs its biological function on the salt response mainly through the roots.

## 3. Discussion

Soybean is an important economic crop which is moderately salt-tolerant [26,56]. Salt salinity is an increasingly serious threat to agricultural production all over the world, and this is the same for soybean farming as well [62,63]. Seeking out new salt-responding candidate genes could provide clues for revealing the salt tolerance mechanism and genetic breeding in soybean. Due to the potential function of CPAs on ion and pH homeostasis, CPA members are likely to participate in salt tolerance in soybean [7]. Herein, the CPA family was systematically analyzed in soybean, especially for their responses to salt stress.

Our study identified 68 *CPA* genes encoding 95 transcripts in soybean. Compared with the previous studies, the present criterion is relatively loose to include as many candidates as possible [23,25,46]. A phylogenetic analysis of *GmCPAs* was performed, comparing *Arabidopsis*, rice, grape, and barrel medic. To be consistent with the previous phylogenetic studies on plant CPA families, the soybean *CPA* family members were divided into *NHX*, *KEA*, and *CHX* subfamilies as well [2,3,5,6]. So far, most knowledge of the *CPA* family has been based on studies in the model plant *Arabidopsis*. For the convenience of comparison, *GmCPAs* were renamed here according to their homologs in *Arabidopsis* and their phylogenic relationship. The gene structure and conserved domain analysis demonstrated that the soybean CPA family shared the similar exon–intron patterns and conserved motif composition as other plants, supporting the present generated phylogenetic tree [16,17,19,20,24]. In addition, the legume-specific CHXs were identified, such as *GmCHX31a* and *GmCHX31b*, which were assumed to function in the legume-specific life processes.

Obviously, the two legumes, soybean and barrel medic, contain much more CPA members than *Arabidopsis*, rice, and grape, especially for the *CHX* subfamily. Most of the *GmCPA* genes came from segmental duplication, indicating that segmental duplication is primarily responsible for the expansion of the *GmCPA* family. Together with the synteny analysis of *GmCPAs* on soybean chromosomes, the expansion of the *CPA* gene family verified the legume-specific whole-genome duplication in evolutionary history [50]. However, the number of *NHX*-type exchangers remained nearly equivalent in the four plant species, suggesting that they are conserved during evolution. Barrel medic contains a similar number of *KEA*-type transporters as *Arabidopsis*, rice, and grape, whereas soybean has twice the amount of *GmKEA*s, demonstrating that there could exist soybean-specific duplication events for the *KEA* subfamily. Meanwhile, the Ka/Ks value of most duplication pairs was less than 1, indicating that GmCPAs experienced a strong purifying selective pressure, which has also been observed in pear, radish, and sorghum [17,19,24]. In general, the Ka/Ks values of Gm*KEA*s were lower than those of Gm*NHX*s and GmCHXs, suggesting that the purifying pressure is stronger in the Gm*KEA* subfamily, consistent with the previous comparative analysis of CPAs in plants [6].

The *cis*-elements in the promoter are the molecular switches of gene transcriptional regulation to govern various biological processes, including development, hormonal fluctuation, and stress responses [25]. Our results revealed that the *cis*-regulatory elements in *GmCPA* promoters mainly contain light-responsive, development-related, stress-responsive, and hormone-responsive elements. The largest type of *cis*-element in *GmCPA*s belongs to the light response elements, similar to previous reports of *CPA*s in maize and *Phyllostachys edulis* [20,21]. However, there has been little evidence to suggest that CPA is involved in light responses until now. In addition, there exist enormous hormone-responsive and stress-responsive elements widely distributed in the promote region of *GmCPA*s. Among the five hormone-responsive *cis*-elements, the numbers of ABA- and MeJA-responsive elements are much higher than those of SA-, auxin-, and gibberellin-responsive elements. As far as we know, ABA and JA are responsible for plant responses to abiotic and biotic stress [64]. In addition, the characterized *GmCPA* members were reported to play a crucial role in salt responses, such as *GmNHX1*, *GmNHX5*, *GmNHX6*, *GmCHX1*/*GmSALT3*, *GsCHX19.3*, and *GmCHX20a* [25,27,28,30,31,32,33,34,36,37,42,44]. Meanwhile, the expression analyses demonstrated that dozens of *GmCPA*s were differentially regulated under salt stress. In conclusion, it is strongly implied that the *CPA* genes are involved in salt responses in soybean. Those *cis*-elements provide clues for a further exploration of their molecular mechanisms in salt tolerance.

Gene expression profiles could provide important hints for the exploration of gene function. It was shown that most of the *GmCPA* genes exhibited a broad expression in all the detected organs, suggesting that they have a wide range of effects on soybean. As salt salinity is a severe threat to soybean production and osmotic stress is the first stage of salt stress [56], the expression patterns of *GmCPA*s were further investigated under salt and osmotic stress. Based on the two sets of transcriptome data, around one third of *GmCPA*s were considered to be deferentially expressed genes, indicating that they play a vital role in salt responses. Among them, the expression levels of 12 candidate genes were verified using qRT-PCR. Some *GmCPA* genes were detected to have similar expression patterns under salt stress and osmotic stress, such as *GmCHX4d*, *GmCHX18a*, *GmCHX18b, GmCHX20a*, and *GmCHX20b1*, suggesting that they might function in a similar mode. However, some *GmCPA*s exhibit different expression patterns under salt stress and osmotic stress, such as *GmCHX22d* and *GmCHX28a*, suggesting that they might play cooperative roles in salt and osmotic tolerance. Some of the selected deferentially expressed genes are duplicated gene pairs, namely *GmCHX18a* and *GmCHX18b*, *GmCHX19b* and *GmCHX19c*, and *GmCHX20a* and *GmCHX20b1*. *GmCHX18a* and *GmCHX18b* have similar expression patterns in both the roots and leaves under salt or osmotic stress, demonstrating that they have a redundant function in salt responses. Likewise, the same situation is applied to the other duplicated genes, *GmNHX2a*/*2b*, *GmNHX5a*/*5b,* and *GmKEA2a/2b*, according to the online transcriptome data. Except for *GmKEA2a/2b*, the other three duplicated gene pairs seem to contain different *cis*-elements, suggesting that they probably function complementally through different pathways. For *GmCHX19b* and *GmCHX19c*, they have similar expression patterns in the leaves under salt or osmotic stress and in the roots under osmotic stress. At the late stage of salt stress, the expression of *GmCHX19b* continued to be highly induced in the roots, whereas the expression of *GmCHX19c* was somehow suppressed as it was observed under osmotic stress. This indicates that *GmCHX19b* and *GmCHX19c* might have a similar effect under osmotic stress or at the early stage of salt stress, whereas *GmCHX19b* plays a special role at the late stage of salt stress, probably for ionic toxicity. The closest homolog of *GmCHX19b* was characterized in wild soybean (*Glycine soja*) as *GsCHX19.3* [37]. *GsCHX19.3* displayed the greatest induction in response to high salinity and carbonate alkaline stress, and this could reduce the Na^+^ concentration in the transgenic *Arabidopsis* lines under salt–alkaline stress. These findings are consistent with our hypothesis for *GmCHX19b*. For *GmCHX20a* and *GmCHX20b1*, their expression patterns are almost opposite, in accordance with our previous study, suggesting that they might provide complementary functions with the opposite effects in soybean under salt stress [36].

To date, there have been some *CPA* members identified for their function in soybean under salt stress, such as *GmCHX1*/*GmSALT3* (renamed as *GmCHX20b* here), *GmCHX20a*, *GsCHX19.3* (the closest homolog of *GmCHX19b* in *Glycine soja*), and the soybean homologs of *NHX*1, *NHX*2, *NHX*5, *NHX*6, and SOS1 [25,27,28,30,31,32,33,34,36,37,42,44], which were present in our lists of differential expression genes under salt stress as well. Among them, the up-regulation of *GmCHX20a* is quite remarkable. To further confirm its function in the salt response, the *GmCHX20a-*overexpressing *Arabidopsis* lines were used here to detect the phenotype and physiological indicators. The *GmCHX20a*-transgenic plants were more sensitive to salt stress with reduced chlorophyll contents, induced MDA contents, and electrical conductivity. The activities of the antioxidant enzymes APX and POD were significantly reduced in the transgenic plants, suggesting that the ectopic expression of *GmCHX20a* brings about more oxidative damage under salt stress. Our previous findings showed that *GmCHX20a* promoted Na^+^ absorption and accumulation under salt treatments according to the results obtained from the BY-2 transformation system [36]. As sodium and potassium are two essential ions involved in salt tolerance [65], their contents and the ratios of Na^+^/K^+^ were checked in the transgenic *Arabidopsis* lines as well. *GmCHX20a* seemed not to alter the ion homeostasis of sodium and potassium too much (Figure S4). It is probable that the regulation of ion homeostasis is more complex in the entire plant, or there are differences in the salt-tolerance mechanisms between soybean and *Arabidopsis*. Altogether, it was verified that *GmCHX20a* plays a negative role in salt tolerance. In the salt-tolerant soybean accessions which contain both *GmCHX20a* and the entire *GmCHX20b* (*GmCHX1*/*GmSALT3*), the two adjacent homologous genes might cooperate with opposite roles to resist salt stress.

In brief, we have systematically investigated the *CPA* family, which is supposed to play important roles in salt tolerance, at the whole genomic level in soybean. Twelve *GmCPA* genes were selected as the salt or osmotic responding candidates. Among them, the most remarkable salt-up-regulated gene *GmCHX20a* was further analyzed for its biological function for salt tolerance. All these results are helpful to reveal the mechanism of salt tolerance in soybean and provide a theoretical basis to improve salt tolerance for soybean breeding.

## 4. Materials and Methods

### 4.1. Identification and Characteristics of CPA Genes in Soybean

The Hidden Markov Model (HMM) profiles of the sodium/hydrogen exchanger family domain (PF00999) were downloaded from the Pfam database (http://pfam.xfam.org/) (accessed on 27 October 2018) [66] and searched as a query against the annotated protein sequences in the *Glycine max* proteome database (Wm82.a2.v1) downloaded from Phytozome v12.0 (http://phytozome.jgi.doe.gov/pz/portal.html) (accessed on 7 February 2020) [67] through HMMER v3.0 (http://hmmer.org) (accessed on 7 February 2020) (E value cutoff level of 1.0). Meanwhile, a BLASTP search was performed via the online tool Phytozome v12.0 using the default settings. The two results were merged, and the retrieved sequences were subsequently examined for the presence of the PF00999 domains in the Interpro database (http://www.ebi.ac.uk/interpro/) (accessed on 17 February 2020) [68].

All the sequences of the genes, transcripts, proteins, and the corresponding putative promoters (2 k bps upstream of the starting translation codon) were downloaded from the Phytozome database. The molecular weights (kDa) and theoretical isoelectric points (pIs) of the putative peptides were calculated in ExPASy (http://web.expasy.org/compute_pi/) (accessed on 27 July 2021). The topology analysis of trans-membrane domains (TMDs) was performed with the TMHMM Server v. 2.0 (http://www.cbs.dtu.dk/services/TMHMM/) (accessed on 27 July 2021) or TMpred (https://embnet.vital-it.ch/software/TMPRED_form.html#opennewwindow) (accessed on 27 July 2021). The subcellular localization was predicted using WoLF PSORT (https://wolfpsort.hgc.jp/) (accessed on 27 July 2021) [69].

### 4.2. Phylogenetic Analysis

Full-length amino acid sequences of the CPA proteins from soybean, *Arabidopsis*, rice, grape, and *Medicago truncatula* were aligned using CLUSTALX version 2.1 [70]. The phylogenetic tree was constructed using MEGA11 software version 11.0.13 with the neighbor-joining method (1000 replicates of bootstrap, Poisson model) [71].

### 4.3. Analysis of Gene Structures and Conserved Domains

The genomic DNA and mRNA sequences of the *CPA* genes were downloaded from the Phytozome database and used to construct gene structures using the online tool of GSDS_2.0_, Gene Structure Display Server 2.0 (http://gsds.cbi.pku.edu.cn/index.php) (accessed on 23 July 2019) [72]. Conserved protein motifs were predicted using the MEME (Multiple Expectation Maximization for Motif Elicitation) program (http://meme-suite.org/tools/meme) (accessed on 31 July 2019) with the default settings [73]. The optimum motif width was set between 6 and 50. The maximum number of motifs was set at 10.

### 4.4. Chromosome Localization and Gene Duplication Analysis

The physical positions of the *CPA* genes on the chromosomes were retrieved from the Phytozome database to draw the map using Mapdraw [74]. The whole genome duplication (WGD)/segmental duplication events were analyzed using the plant genome duplication database (PGDD, http://chibba.agtec.uga.edu/duplication/index/downloads) (accessed on 3 March 2020) [75]. In addition, duplicated genes were also identified in the terminal nodes of the phylogenetic tree of the soybean CPA proteins with a strong bootstrap value (>85%) and high sequence similarity (>70%) [47]. Generally, the duplicated genes on different chromosomes were defined as the segmental duplicates, and those located within 20 loci on the same chromosome were set as the tandem duplicates [76]. The image of chromosomal locations and the duplication events was generated in the R program version 3.6.2 (https://cran.r-project.org/) (accessed on 18 October 2023) with the package “circlize” [77]. The synonymous substitution rate (Ks) and non-synonymous substitution rate (Ka) were estimated by DnaSP v5 [78]. The ratio of Ka/Ks was determined for the mode of selection (Ka/Ks > 1: positive selection; Ka/Ks = 1: neutral evolution; Ka/Ks < 1: purify selection) [79]. For each duplication event, the estimated divergence time was calculated as Ks/(2 × 6.1 × 10^−9^) × 10^−6^ Mya (million years ago) based on the clock-like rate (λ) of 6.1 × 10^−9^ synonymous substitutions per site per year [80].

### 4.5. Analysis of cis-Acting Regulatory Elements in Promoter

The promoter sequences (the 2000 bp region genomic DNA sequences upstream from the ATG start codon) of the *GmCPA* genes were downloaded from the Phytozome database. The *cis*-acting regulatory elements were analyzed using PlantCARE (http://bioinformatics.psb.ugent.be/webtools/plantcare/html/) (accessed on 27 January 2020) [81], and the visualization was generated by TBtools version 1.09852 [82].

### 4.6. Digital Expression Analysis of GmCPAs in Different Tissues or under Abiotic Stress

The transcriptional data from fourteen tissues, including young leaf, flower, 1 cm pod, pod shell 10 DAF (day after flowering), pod shell 14 DAF, seed 10 DAF, seed 14 DAF, seed 21 DAF, seed 25 DAF, seed 28 DAF, seed 35 DAF, seed 42 DAF, root, and nodule, were downloaded at Soybase (http://www.soybase.org/soyseq/) (accessed on 6 April 2020) [54]. In addition, two published RNA-seq data of soybean seedlings under salt or dehydration stress were downloaded as well to investigate the potential function of *GmCPAs* in salt responses. The Illumina sequencing data (NCBI GEO database: *GSE57252*, BioProject ID number: PRJNA246058) were obtained from the soybean roots treated with 100 mM NaCl or dehydration at three time points (1 h, 6 h, and 12 h) or the roots without salt treatment (control) [57]). Another one was taken from the soybean leaves or roots treated with 0.9% NaCl (*w*/*w*; ~150 mM) at six time points (1 h, 2 h, 4 h, 8 h, 12 h, 24 h, and 48 h), respectively [58]. The samples were taken from both the cultivated soybean (*Glycine max*) accession C08 (variety name: Union, salt-sensitive) and the wild soybean (*Glycine soja*) accession W05 (variety name: Mengjin1, salt-tolerant). The values of normalized Reads/Kb/Million (RPKM) were log2-transformed and displayed in a heatmap, which was generated with R version 3.6.2 using the pheatmap package (https://cran.r-project.org/web/packages/pheatmap/) (accessed on 18 October 2023).

### 4.7. Gene Expression Analysis of GmCPAs under Salt or Osmotic Treatments

The soybean seedlings grew in vermiculite with water for one week after germination, followed by being transferred to a hydroponic system with half-strength Hoagland’s nutrient solution, as previously described [47]. With the opening of the first trifoliate, the seedlings were treated with half-strength Hoagland’s solution containing 0.9% NaCl or 5% PEG for different time points (0 h, 1 h, 6 h, 12 h, and 24 h). The total RNA was extracted, respectively, from the roots and leaves of the treated seedlings, which were collected and frozen in liquid nitrogen. The expression levels of the selected *GmCPA* genes were detected using quantitative real-time PCR (qRT-PCR) with the PrimerScript^TM^ one step RT-PCR kit (TaKaRa Biotechnology Co., Ltd., Dalian, China) according to the manufacturer’s protocol. The used primers are listed in Table S6. The 2^−ΔΔCt^ method was used to calculate the relative gene expression level [83]. The *GmELF1b* gene was regarded as an endogenous control for normalization [84]. Three independent biological replicas were performed for the qRT-PCR analysis.

### 4.8. Salt Tolerance Assay on the GmCHX20a-Overexpressing Arabidopsis thaliana Plants

The *GmCHX20a* gene was cloned from the cultivated soybean (*Glycine max*) accession C08 (variety name: Union, salt-sensitive) and the wild soybean (*Glycine soja*) accession W05 (variety name: Mengjin1, salt-tolerant), respectively. They were transformed into *Arabidopsis thaliana* (ecotype: Columbia-0, Col-0) using the floral dip method to obtain the single-insertion homozygotes in T3 generations, as described previously [36]. The expression of *GmCHX20a* was verified in the transgenic lines using qRT-PCR, and the three independent lines of each transformation were selected for further investigation. The *Arabidopsis* plants were grown under a 16 h light/8 h dark cycle at 21 °C with around 75% humidity. The 7-day seedlings growing on the 1/2 MS solid media were transferred to the fresh 1/2 MS media with or without 150 mM NaCl for 14 days to observe the phenotype. The 10-day seedlings growing in a 1/10 MS hydroponic system were transferred to the fresh 1/10 MS solution with or without 100 mM NaCl for 24 h treatment to detect the antioxidant enzyme activities, and for a 3-day treatment to measure the chlorophyll contents, electrical conductivity (EC), and malondialdehyde (MDA) contents. The activities of catalase (CAT), ascorbate peroxidase (APX), superoxide dismutase (SOD), peroxidase (POD), and the total chlorophyll contents were determined as described previously [59]. The MDA content was measured using the thiobarbituric acid (TBA) method with minor modifications [85]. A total of 50 mg of plant leaves was homogenized in 1 mL 5% trichloroacetic acid (TCA) solution and centrifuged at 3000 rpm for 10 min. The supernatant was added to 0.67% TBA in 10% TCA of the same volume. The mixture was boiled for 30 min and then cooled in ice. Followed by centrifugation, the MDA concentration was calculated by monitoring the absorbance at 450, 532, and 600 nm. The formula is C_MDA_(μM) = 6.45 × (D_532_ − D_600_) − 0.56 × D_450_. The relative damage rate was determined based on the electrical conductivity (EC) method [86]. A total of 50 mg of plant leaves was rinsed with deionized water to remove surface contamination. Then, the leaf samples were soaked in 20 mL deionized water with 10 min evacuation and stayed at room temperature for 20 min. Half of the supernatant was taken out and diluted with 10 mL deionized water to measure the initial electrical conductivity (E1) using a conductivity meter. The left samples with the leaves were boiled for 20 min after adding 10 mL deionized water. After cooling to room temperature, the second reading (E2) was measured. The damage rate was calculated as E1/E2. The relative chlorophyll, MDA content, and damage rate were taken to compare the value from the salt-treated plants with those from their respective controls under normal conditions. The contents of sodium and potassium ions were determined by the flame atomic absorption spectrophotometer (WFX-130A, Beijing Beifen-Ruili Analytical Instrument (Group) Co., Ltd., Beijing, China) as previously described [36].

### 4.9. Subcellular Localization by Transient Expression in Onion Epidermis

The full-length coding sequences of *GmCHX20a* were cloned into the Gateway binary vector pGWB5 via Gateway^TM^ method (Invitrogen) [87]. GFP was fused in the N terminal of *GmCHX20a* under the control of the cauliflower mosaic virus 35S promoter. After being coated with gold particles, the constructs were bombarded into the epidermal cells of onion (Bio-Rad PDS-1000/He system, Hercules, CA, USA). The onion sections were imaged using a Leica SP8 X inverted confocal microscope with an Argon laser (Leica, Wetzlar, Germany) for GFP signals (excitation: 488 nm, emission: 510–525 nm). To generate plasmolysis, the samples were incubated in 25% sucrose for 5 min.

### 4.10. Statistical Analysis

The results were presented as the mean ± standard deviation (SD) of at least three replicas. Statistical significance differences were determined using the Statistical Package for Social Sciences (version 19.0, SPSS Inc. Chicago, IL, USA) at the level of *p* < 0.05 or *p* < 0.01.

## Data Availability

Data are contained within the article.

## Supplementary Materials

The following supporting information can be downloaded at: https://www.mdpi.com/article/10.3390/ijms242316560/s1.
