# Supplementary material for: Genome-Wide Analysis of Cation/Proton Antiporter Family in Soybean (Glycine max) and Functional Analysis of GmCHX20a on Salt Response
Source: Int J Mol Sci. 2023 Nov 21;24(23):16560. doi: 10.3390/ijms242316560 (PMC10705888; doi:10.3390/ijms242316560)
Supplement: Supplementary file 1 [file ijms-24-16560-s001.zip › supplementarypv1/ijms-2699473-caption of supplementary.pdf]

Figure S1: Chromosomal locations of the *GmCPA* genes;
Figure S2: Heat map exhibiting the expression profiles of *GmCPA*s under salt stress;
Figure S3: The effects of the *GmCHX20a* overexpression on the H_2O_2 contents under salt stress by DAB staining;
Figure S4: Na^+ content and Na^+/K^+ ratio in Arabidopsis transgenic lines;
Table S1: The list of the CPA family members in soybean;
Table S2: Conserved motifs of GmCPA predicted by MEME;
Table S3: Divergence between *GmCPA* gene pairs;
Table S4: *Cis*-acting regulatory elements in the promoter regions of the *GmCPA* genes;
Table S5: Number of *cis*-acting elements in the promoter regions of the *GmCPA* genes;
Table S6: Primers used in this study.
