# Supplementary material for: Genome-Wide Analysis of Cation/Proton Antiporter Family in Soybean (Glycine max) and Functional Analysis of GmCHX20a on Salt Response
Source: Int J Mol Sci. 2023 Nov 21;24(23):16560. doi: 10.3390/ijms242316560 (PMC10705888; doi:10.3390/ijms242316560)
Supplement: Supplementary file 1 [file ijms-24-16560-s001.zip › supplementarypv1/suplementary materials20231106/Figure S2-cpaxzx_复制.pdf]

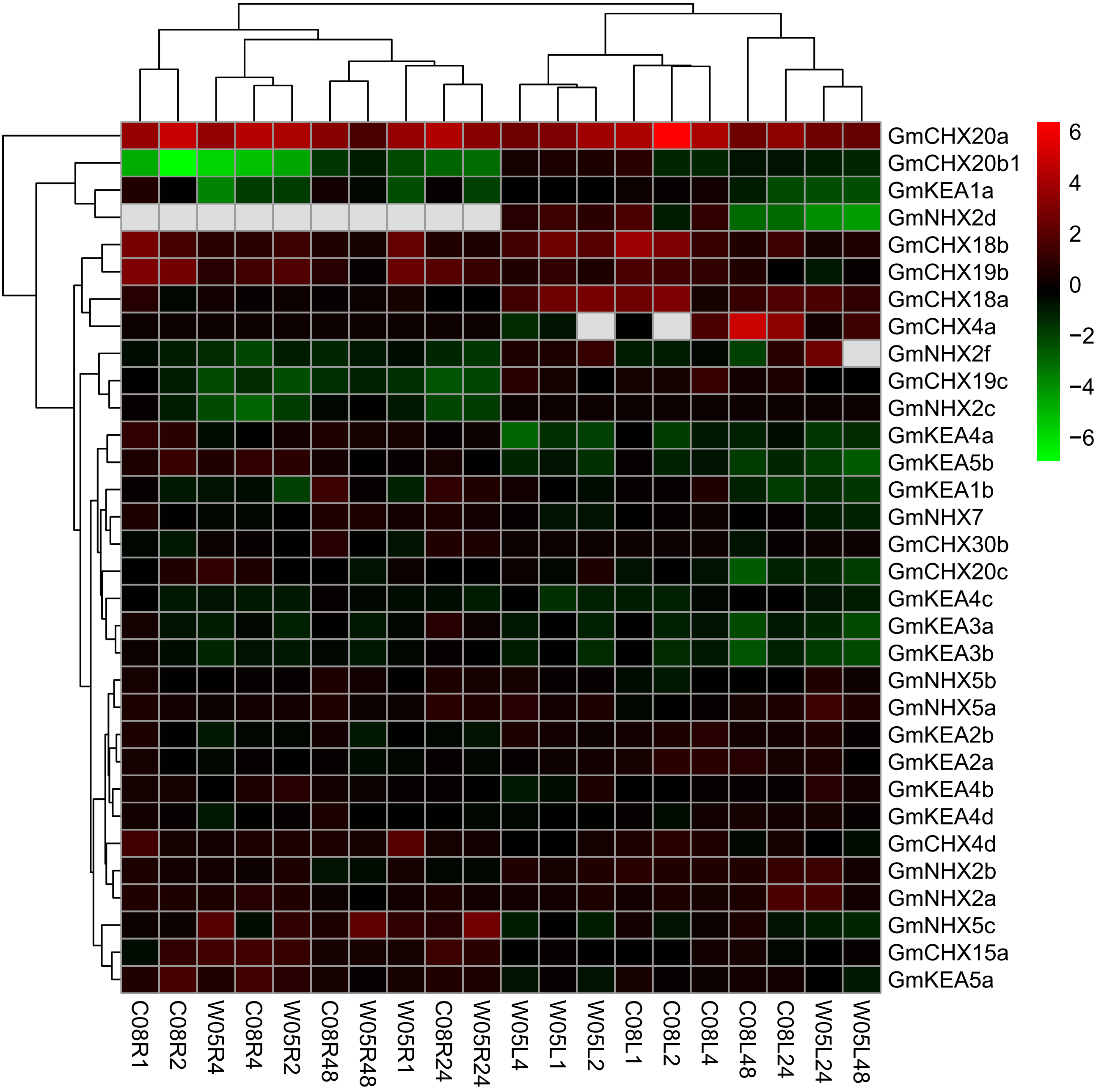

Figure S2. Heat map exhibiting the expression profiles of *GmCPA*s under salt stress. The log2 values of the relative expression data compared with the controls were presented by different colors. The color scale was shown on the right. Green indicated reduced expression and red up-regulated expression. C08L1, C08L2, C08L4, C08L24, and C08L48 presented for the leaves collected from the cultivated soybean C08 at 1 h, 2 h, 4 h, 24 h, and 48 h after salt treatments, respectively. W05L1, W05L2, W05L4, W05L24, and W05L48 presented for the leaves collected from the wild soybean W05 at 1 h, 2 h, 4 h, 24 h, and 48 h after salt treatments, respectively. C08R1, C08R2, C08R4, C08R24, and C08R48 presented for the roots collected from C08 at 1 h, 2 h, 4 h, 24 h, and 48 h after salt treatments, respectively. W05R1, W05R2, W05R4, W05R24, and W05R48 presented for the leaves collected from W05 at 1 h, 2 h, 4 h, 24 h, and 48 h after salt treatments, respectively.
