# Supplementary material for: Genome-Wide Analysis of Cation/Proton Antiporter Family in Soybean (Glycine max) and Functional Analysis of GmCHX20a on Salt Response
Source: Int J Mol Sci. 2023 Nov 21;24(23):16560. doi: 10.3390/ijms242316560 (PMC10705888; doi:10.3390/ijms242316560)
Supplement: Supplementary file 1 [file ijms-24-16560-s001.zip › supplementarypv1/suplementary materials20231106/Figure S4GmCHX20aNAK_legend_复制.pdf]

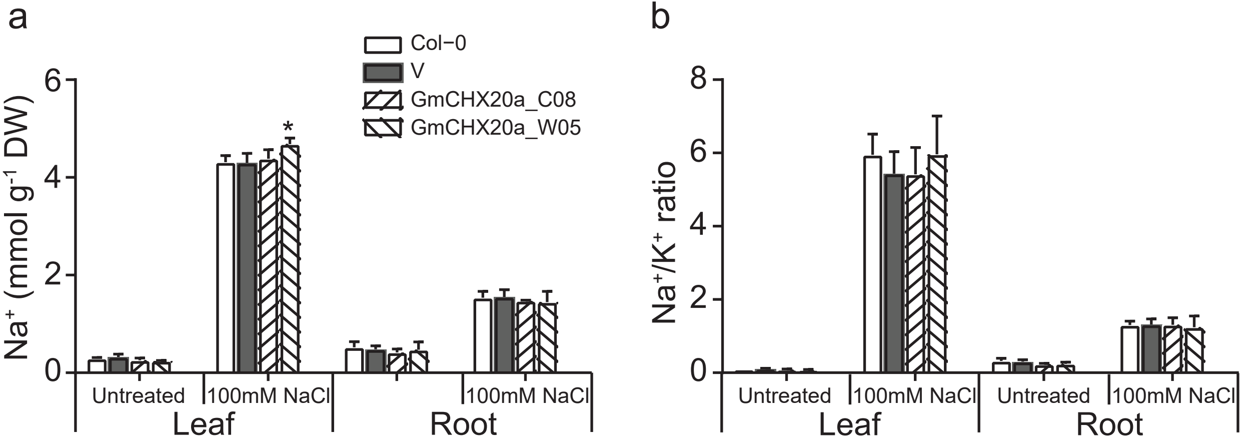

Figure S4. Na^+ content and Na^+/K^+ ratio in Arabidopsis transgenic lines. The aerial parts and roots of seedlings treated with or without 100mM NaCl for 7 days were harvested separately. Then, the Na^+ content (a) and Na^+/K^+ ratio (b) were detected. Values are mean ± SD. The asterisk marks represented for significant differences in comparison to the wild type (Col−0) and the mock line (transformed with the empty vector, denoted as V) at $p < 0.05$ (one-way ANOVA). GmCHX20a_C08 and GmCHX20a_W05 are the transgenic line expressing GmCHX20a from C08 to W05, respectively.
