# Supplementary material for: Genome-Wide Analysis of Cation/Proton Antiporter Family in Soybean (Glycine max) and Functional Analysis of GmCHX20a on Salt Response
Source: Int J Mol Sci. 2023 Nov 21;24(23):16560. doi: 10.3390/ijms242316560 (PMC10705888; doi:10.3390/ijms242316560)
Supplement: Supplementary file 1 [file ijms-24-16560-s001.zip › supplementarypv1/suplementary materials20231106/Table S2-GmCPAmotifmeme.pdf]

Table S2. Conserved motifs of GmCPA predicted by MEME.

Motif	E-value	Sites	Width	Sequence
1	1.2e-775	43	41	VSYRVAVJFIGGPDDREALAYAWRMAEHPGVK LTVVRFVLS
2	7.8e-741	43	41	GAVSVQTYTAISPYSTMHEDICNLALDKRASJII LPFHKQW
3	1.1e-647	60	37	ALSVTSFPVVARILAELKJLNTELGRLAJSAALIN DV
4	6.3e-471	43	21	RALNQNVLEHAPCSVGILVDR
5	9.60e-288	11	50	YIIRHSTDREVALMVLMAYLSYMLAELFYLSGIL TVFFCGIVMSHYTWHN
6	8.80e-287	44	17	SECPELGVIGDLLASSD
7	2.1e-444	56	31	GMPLKDGLALGLLLNTKGLVELVVLNIAKBQ
8	2.1e-419	42	28	ELRILACIHNPRNIPSMINLLEASNPTR
9	4.20e-221	60	15	FGLIFFLFLVGLEMD
10	3.70e-193	58	15	LGAFVFGLIIPEGPP
