# Supplementary material for: Genome-Wide Analysis of Cation/Proton Antiporter Family in Soybean (Glycine max) and Functional Analysis of GmCHX20a on Salt Response
Source: Int J Mol Sci. 2023 Nov 21;24(23):16560. doi: 10.3390/ijms242316560 (PMC10705888; doi:10.3390/ijms242316560)
Supplement: Supplementary file 1 [file ijms-24-16560-s001.zip › supplementarypv1/suplementary materials20231106/Table S3-DivergenceGmCPA.pdf]

Table S3. Divergence between *GmCPA* gene pairs.

Gene pair	locus	Ks	Ka	Ka/Ks	Date (MYA)	Duplication event
GmCHX18a/ GmCHX18b	Glyma.01g223600/ Glyma.11G019900	0.12	0.02	0.14	9.43	segmental
GmKEA1a/ GmKEA1b	Glyma.03g014000/ Glyma.07G073700	0.10	0.02	0.22	8.03	segmental
GmKEA1a/ GmKEA2b	Glyma.03G014000/ Glyma.09G262000	0.22	0.19	0.83	18.37	segmental
GmKEA1a/ GmKEA2a	Glyma.03G014000/ Glyma.18G230100	0.20	0.18	0.88	16.53	segmental
GmCHX20a/ GmCHX20b2	Glyma.03G171500/ Glyma.03G171700	2.13	0.38	0.18	174.84	tandem
GmCHX20a/ GmCHX20c	Glyma.03G171500/ Glyma.13G131200	1.05	0.21	0.20	86.41	segmental
GmCHX22a/ GmCHX22g	Glyma.03g233700/ Glyma.19G230900	0.99	0.34	0.34	81.41	segmental
GmNHX2d/ GmNHX2b	Glyma.03g243000/ Glyma.10G158700	0.44	0.12	0.27	36.22	segmental
GmNHX2d/ GmNHX2e	Glyma.03G243000/ Glyma.19G240500	0.06	0.02	0.32	5.23	segmental
GmNHX2d/ GmNHX2a	Glyma.03G243000/ Glyma.20G229900	0.45	0.12	0.27	36.67	segmental
GmCHX22b/ GmCHX22c	Glyma.04G187400/ Glyma.06G178500	0.28	0.16	0.58	22.82	segmental
GmCHX15a/ GmCHX15d	Glyma.04g212200/ Glyma.05G192400	0.70	0.25	0.36	57.35	segmental
GmCHX15a/ GmCHX15b	Glyma.04G212200/ Glyma.06G154200	0.21	0.05	0.22	17.22	segmental
GmCHX3a/ GmCHX3b	Glyma.05G153700/ Glyma.18G001600	0.32	0.39	1.19	26.52	segmental
GmCHX15d/ GmCHX15b	Glyma.05g192400/ Glyma.06G154200	0.28	0.38	1.39	22.58	segmental
GmKEA4a/ GmKEA4b	Glyma.05g222500/ Glyma.08G029300	0.10	0.02	0.17	7.88	segmental
GmKEA1b/ GmKEA2b	Glyma.07g073700/ Glyma.09G262000	0.44	0.12	0.27	36.33	segmental
GmKEA1b/ GmKEA2a	Glyma.07G073700/ Glyma.18G230100	0.14	0.19	1.35	11.67	segmental
GmKEA3a/ GmKEA3b	Glyma.07g184800/ Glyma.08G064600	0.10	0.02	0.21	8.12	segmental
GmNHX2f/ GmNHX2c	Glyma.07g251900/ Glyma.17G022500	0.09	0.01	0.10	7.20	segmental

Gene pair	locus	Ks	Ka	Ka/Ks	Date (MYA)	Duplication event
GmNHX5c/ GmNHX5b	Glyma.07g270400/ Glyma.09G018200	0.61	0.08	0.13	49.78	segmental
GmNHX5c/ GmNHX5a	Glyma.07G270400/ Glyma.15G124100	0.53	0.08	0.15	43.04	segmental
GmNHX5b/ GmNHX5a	Glyma.09g018200/ Glyma.15G124100	0.12	0.01	0.06	9.70	segmental
GmKEA5a/ GmKEA5b	Glyma.09g152300/ Glyma.16G203200	0.07	0.01	0.15	5.98	segmental
GmCHX28c/ GmCHX28a	Glyma.09g228400/ Glyma.12G008100	0.17	0.07	0.40	13.61	segmental
GmKEA2b/ GmKEA2a	Glyma.09g262000/ Glyma.18G230100	0.11	0.02	0.20	8.70	segmental
GmCHX28b/ GmCHX1	Glyma.10g089900/ Glyma.12G241200	1.04	0.83	0.79	85.25	segmental
GmNHX2b/ GmNHX2e	Glyma.10g158700/ Glyma.19G240500	0.48	0.12	0.24	39.67	segmental
GmNHX2b/ GmNHX2a	Glyma.10G158700/ Glyma.20G229900	0.10	0.01	0.09	8.48	segmental
GmCHX14b/ GmCHX29a	Glyma.11G163600/ Glyma.11G234000	1.13	0.77	0.68	92.74	segmental
GmCHX14b/ GmCHX30a	Glyma.11G163600/ Glyma.14G200000	1.32	0.72	0.54	108.11	segmental
GmCHX14b/ GmCHX29b	Glyma.11G163600/ Glyma.18G023000	0.94	0.82	0.87	76.80	segmental
GmCHX14b/ GmCHX14a	Glyma.11G163600/ Glyma.18G056900	0.14	0.06	0.40	11.79	segmental
GmCHX22f/ GmCHX22g	Glyma.11g229400/ Glyma.18G027900	0.19	0.05	0.27	15.30	segmental
GmCHX29a/ GmCHX30a	Glyma.11g234000/ Glyma.14G200000	1.05	0.74	0.70	85.93	segmental
GmCHX29a/ GmCHX29b	Glyma.11G234000/ Glyma.18G023000	0.15	0.09	0.60	12.16	segmental
GmCHX29a/ GmCHX32	Glyma.11G234000/ Glyma.18G056800	1.90	0.65	0.34	155.82	segmental
GmCHX19c/ GmCHX19b	Glyma.13g116400/ Glyma.17G043400	0.09	0.03	0.28	7.45	segmental
GmCHX4c/ GmCHX4b	Glyma.14G037600/ Glyma.14G037700	0.01	0.00	0.44	0.77	tandem
GmCHX4c/ GmCHX4d	Glyma.14G037600/ Glyma.14G037800	0.21	0.09	0.41	17.31	tandem
GmCHX4c/ GmCHX4e	Glyma.14G037600/ Glyma.20G045400	0.41	0.52	1.27	33.29	segmental

Gene pair	locus	Ks	Ka	Ka/Ks	Date (MYA)	Duplication event
GmCHX4b/ GmCHX4d	Glyma.14G037700/ Glyma.14G037800	0.21	0.09	0.41	17.49	tandem
GmKEA4d/ GmKEA4c	Glyma.14g093900/ Glyma.17G229700	0.08	0.01	0.10	6.36	segmental
GmCHX30a/ GmCHX30b	Glyma.14G200000/ Glyma.14G200100	0.26	0.13	0.50	21.54	tandem
GmCHX30a/ GmCHX29b	Glyma.14G200000/ Glyma.18G023000	0.89	0.80	0.90	73.09	segmental
GmCHX30a/ GmCHX32	Glyma.14g200000/ Glyma.18G056800	0.89	0.79	0.89	72.55	segmental
GmCHX29b/ GmCHX32	Glyma.18g023000/ Glyma.18G056800	1.87	0.68	0.36	153.43	segmental
GmCHX31a/ GmCHX31b	Glyma.18G023700/ Glyma.18G023800	0.48	0.47	0.97	39.41	tandem
GmNHX2e/ GmNHX2a	Glyma.19g240500/ Glyma.20G229900	0.49	0.11	0.23	40.15	segmental
