# Supplementary material for: Genome-Wide Analysis of Cation/Proton Antiporter Family in Soybean (Glycine max) and Functional Analysis of GmCHX20a on Salt Response
Source: Int J Mol Sci. 2023 Nov 21;24(23):16560. doi: 10.3390/ijms242316560 (PMC10705888; doi:10.3390/ijms242316560)
Supplement: Supplementary file 1 [file ijms-24-16560-s001.zip › supplementarypv1/suplementary materials20231106/Table S5-number of cis.pdf]

Table S5. Number of *cis*-acting elements in the promoter regions of the GmCPA genes.

Group	Name	GeneID	light responsive element	development - related element	stress - responsive element	MeJA - responsive element	ABA - responsive element	SA- responsive element	auxin- responsive element	gibberellin -responsive element
NHXIa	GmNHX2a	Glyma.20G229900	9	0	3	4	3	0	0	0
	GmNHX2b	Glyma.10G158700	13	2	0	0	2	1	1	0
	GmNHX2d	Glyma.03G243000	11	1	8	0	1	0	1	0
	GmNHX2e	Glyma.19G240500	15	1	3	0	0	0	0	0
	GmNHX2f	Glyma.07G251900	18	3	1	6	2	0	0	1
	GmNHX2c	Glyma.17G022500	15	3	4	0	3	0	0	0
	GmNHX3	Glyma.08G324200	17	0	3	4	3	1	2	0
NHXIb	GmNHX5a	Glyma.15G124100	14	0	5	0	1	0	1	0
	GmNHX5b	Glyma.09G018200	15	0	4	4	3	0	0	0
	GmNHX5c	Glyma.07G270400	2	4	0	0	0	0	0	1
NHXII	GmNHX7	Glyma.08G092000	14	2	3	4	0	1	0	0
KEAI	GmKEA1a	Glyma.03G014000	10	1	4	0	0	1	1	0
	GmKEA1b	Glyma.07G073700	13	0	2	2	0	0	1	1
	GmKEA2a	Glyma.18G230100	11	2	6	0	2	1	0	0
	GmKEA2b	Glyma.09G262000	16	2	4	0	3	3	0	0
	GmKEA3a	Glyma.07G184800	18	3	6	0	2	0	0	1
	GmKEA3b	Glyma.08G064600	15	2	1	2	0	0	1	0
KEAII	GmKEA4a	Glyma.05G222500	9	1	5	0	2	0	0	0
	GmKEA4b	Glyma.08G029300	19	0	1	0	2	0	0	0
	GmKEA4c	Glyma.17G229700	19	1	2	2	0	0	1	0
	GmKEA4d	Glyma.14G093900	19	0	4	2	0	1	1	0
	GmKEA5a	Glyma.09G152300	22	2	2	2	4	0	0	1
	GmKEA5b	Glyma.16G203200	13	1	0	0	4	1	0	3

Group	Name	GeneID	light responsive element	development - related element	stress - responsive element	MeJA - responsive element	ABA - responsive element	SA- responsive element	auxin- responsive element	gibberellin -responsive element
CHXI	GmCHX1	Glyma.12G241200	12	3	4	2	3	1	0	0
	GmCHX28a	Glyma.12G008100	16	3	6	0	1	1	0	1
	GmCHX28c	Glyma.09G228400	12	1	3	2	0	1	1	0
	GmCHX28b	Glyma.10G089900	24	2	1	0	0	1	2	0
CHXV	GmCHX25a	Glyma.18G057400	18	2	4	2	2	0	1	2
	GmCHX25b	Glyma.11G164200	14	1	3	0	0	0	0	0
CHXIII	GmCHX29a	Glyma.11G234000	13	1	2	2	3	1	0	2
	GmCHX29b	Glyma.18G023000	17	0	3	2	4	0	0	3
	GmCHX30a	Glyma.14G200000	18	1	5	2	0	0	0	0
	GmCHX30b	Glyma.14G200100	8	2	1	2	0	0	2	0
CHXII	GmCHX3a	Glyma.05G153700	5	1	5	0	1	1	0	3
	GmCHX3b	Glyma.18G001600	10	0	6	2	1	1	0	1
	GmCHX3c	Glyma.02G218800	11	0	5	0	0	1	0	0
	GmCHX4e	Glyma.20G045400	11	1	4	0	0	1	0	1
	GmCHX4d	Glyma.14G037800	20	2	3	2	3	1	0	0
	GmCHX4c	Glyma.14G037600	21	2	4	0	2	0	0	0
	GmCHX4b	Glyma.14G037700	11	3	3	2	2	2	1	0
	GmCHX4a	Glyma.09G128600	16	2	5	0	2	0	1	1
CHXIII	GmCHX32	Glyma.18G056800	15	0	4	2	1	0	0	0
	GmCHX14a	Glyma.18G056900	10	2	7	0	0	0	4	1
	GmCHX14b	Glyma.11G163600	12	3	1	4	1	0	0	0
	GmCHX31a	Glyma.18G023700	10	1	2	2	3	0	0	1
	GmCHX31b	Glyma.18G023800	17	2	2	4	1	2	0	0

Group	Name	GeneID	light responsive element	development - related element	stress - responsive element	MeJA - responsive element	ABA - responsive element	SA- responsive element	auxin- responsive element	gibberellin -responsive element
CHXIVc	GmCHX20b2	Glyma.03G171700	8	3	4	4	0	1	0	0
	GmCHX20a	Glyma.03G171500	12	3	2	6	3	0	1	0
	GmCHX20b1	Glyma.03G171600	12	0	2	2	0	0	0	0
	GmCHX20c	Glyma.13G131200	13	3	4	2	4	1	0	1
CHXIVb	GmCHX18a	Glyma.01G223600	19	1	3	0	3	0	0	1
	GmCHX18b	Glyma.11G019900	20	1	1	2	1	0	0	0
	GmCHX19a	Glyma.13G081300	13	4	3	0	6	0	0	0
	GmCHX19b	Glyma.17G043400	17	1	2	2	4	0	1	1
	GmCHX19c	Glyma.13G116400	17	2	4	2	7	2	1	0
CHXIVa	GmCHX15a	Glyma.04G212200	15	0	2	2	3	0	1	0
	GmCHX15b	Glyma.06G154200	13	2	7	2	7	0	2	2
	GmCHX15d	Glyma.05G192400	18	3	1	0	3	0	1	0
	GmCHX15c	Glyma.15G066300	20	3	7	0	5	0	1	1
CHXIVd	GmCHX22a	Glyma.03G233700	14	0	6	0	2	0	0	1
	GmCHX22h	Glyma.19G230900	14	1	3	0	1	0	0	1
	GmCHX22d	Glyma.08G057800	15	2	2	6	5	0	1	2
	GmCHX22e	Glyma.10G131500	10	1	0	4	0	1	0	2
	GmCHX22b	Glyma.04G187400	19	4	5	0	8	0	1	0
	GmCHX22c	Glyma.06G178500	7	1	4	8	2	0	0	0
	GmCHX22f	Glyma.11G229400	13	3	2	0	0	2	0	0
	GmCHX22g	Glyma.18G027900	14	0	2	2	2	1	1	0
	GmCHX23	Glyma.16G039700	16	0	5	2	3	1	1	1
