# Supplementary material for: Genome-Wide Analysis of Cation/Proton Antiporter Family in Soybean (Glycine max) and Functional Analysis of GmCHX20a on Salt Response
Source: Int J Mol Sci. 2023 Nov 21;24(23):16560. doi: 10.3390/ijms242316560 (PMC10705888; doi:10.3390/ijms242316560)
Supplement: Supplementary file 1 [file ijms-24-16560-s001.zip › supplementarypv1/suplementary materials20231106/Table S6-GmCPAprimers.pdf]

Table S6. Primers used in this study.

**Name***	**Sequences (5' to 3')**	**Gene**	**Purpose**
JO198F	TCACTGTGCCCTTTCTTGGA	GmNHX2e (Glyma.19G240500)	qRT-PCR
JO199R	CTACCACCAAAAACCGGACG		
JO194F	CAAGCACTTCCCAAACGTGA	GmKEA1b (Glyma.07g073700)	qRT-PCR
JO195R	TAGTCGCTGCAATCTCCGAT		
JO196F	AGTATTTGGCCCATGCTTGC	GmKEA3a (Glyma.07g184800)	qRT-PCR
JO197R	AGTGAAGTCCCTGCAACAGT		
JO192F	TCACGTGTTGTGCCATGAAG	GmCHX4d, (Glyma.14g037800)	qRT-PCR
JO193R	AGAAACCATCCGTTTTGGGC		
JO200F	CCTCTCGTGACTGCTGTGTA	GmCHX18a, (Glyma.01g223600)	qRT-PCR
JO201R	CACCCTTCCGGATTCCTCTT		
JO182F	TTGTTGATCGTGGGCTTGGT	GmCHX18b, (Glyma.11G019900)	qRT-PCR
JO183R	AGCAATCTGATGCCAGGGTG		
JO184F	CCTCTTTGGGGCTTTTGTGC	GmCHX19b, (Glyma.17G043400)	qRT-PCR
JO185R	TGCCCAAGAAAGTCCTCCAC		
JO202F	CTAGCTTGCTTCCACACCAC	GmCHX19c, (Glyma.13g116400)	qRT-PCR
JO203R	ATTCTTGCGTGCCTTGTGAA		
JO113F	CGTATGCATCATCTTCTTTGG	*GmCHX20a, (Glyma.03G171500)*	qRT-PCR
JO114R	GGTCATCCTTTTCAACAAACC		
JO180F	TATGGGTGCTCTTCTCAGGG	*GmCHX20b1,* (Glyma.03G171600*)*	qRT-PCR
JO181R	GGGTAACACGTCTTGTTCGTG		
JO186F	CAATACTCGCCAAGCCACAG	GmCHX22d, (Glyma.08G057800)	qRT-PCR
JO187R	GGGTTGGCTACTAGGCTTCT		
JO188F	CTCCTTATCACCAAGGGCCA	GmCHX28a, (Glyma.12G008100)	qRT-PCR
JO189R	TCCAGTAATTGGAGGGCGTT		
JO190F	TCATCGTTTGGCGCTTCAAT	GmCHX28b, (Glyma.10G089900)	qRT-PCR
JO191R	GCACAATACTGGTGTCAGCC		
GmELF1bF	CCACTGCTGAAGAAGATGATGATG	Glyma.02G276600, Glyma.14G039100	qRT-PCR
GmELF1bR	AAGGACAGAAGACTTGCCACTC		
JO138F	AATCTAGAATGCATATGGCGTTGA ACAT	*GmCHX20a*	cDNA cloning
JO139R	GGCTCGAGTTACACTACGTTGTCG TTTG		
Act2F	ACATTGTGCTCAGTGGTGGA	*AtACT2 (At3g18780)*	qRT-PCR
Act2R	TCATACTCGGCCTTGGAGAT		

*F:forward primer; R: reverse primer.
