# Supplementary figures and images for: Genome-Wide Analysis of Cation/Proton Antiporter Family in Soybean (Glycine max) and Functional Analysis of GmCHX20a on Salt Response
Source: Int J Mol Sci. 2023 Nov 21;24(23):16560. doi: 10.3390/ijms242316560 (PMC10705888; doi:10.3390/ijms242316560)

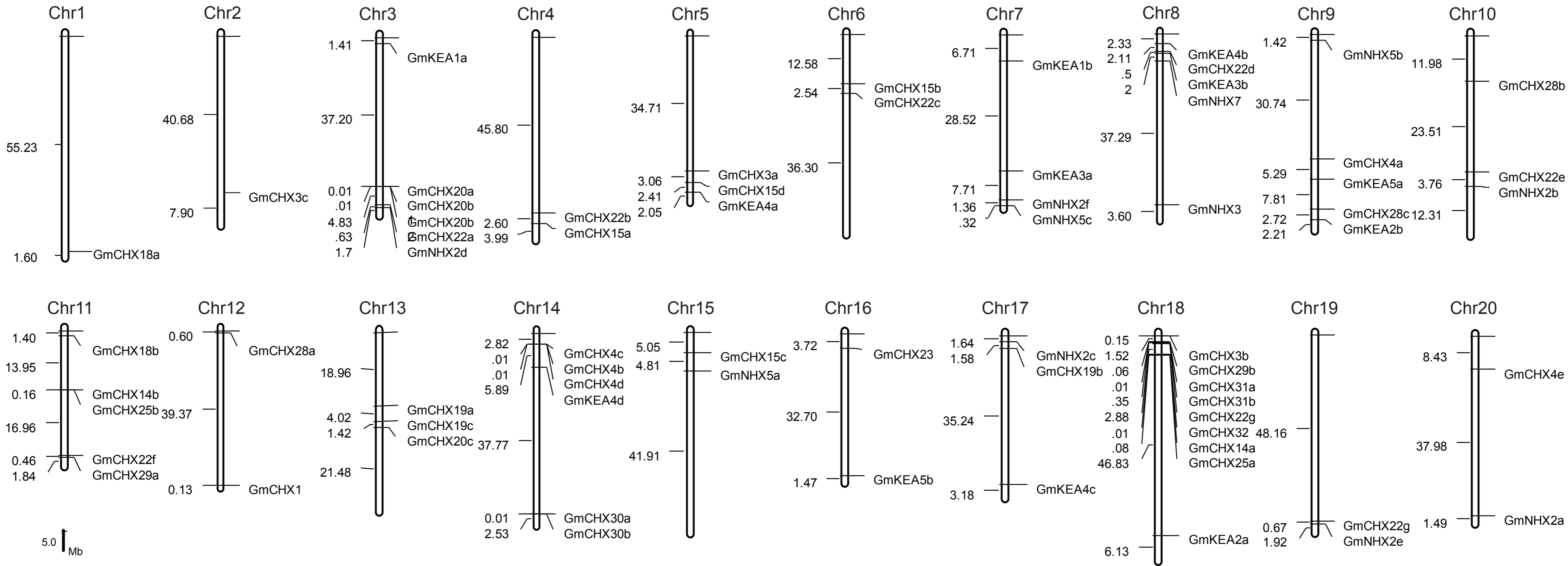

Supplement: Supplementary file 1 [file ijms-24-16560-s001.zip › supplementarypv1/suplementary materials20231106/Figure S1-cpachr.pdf]

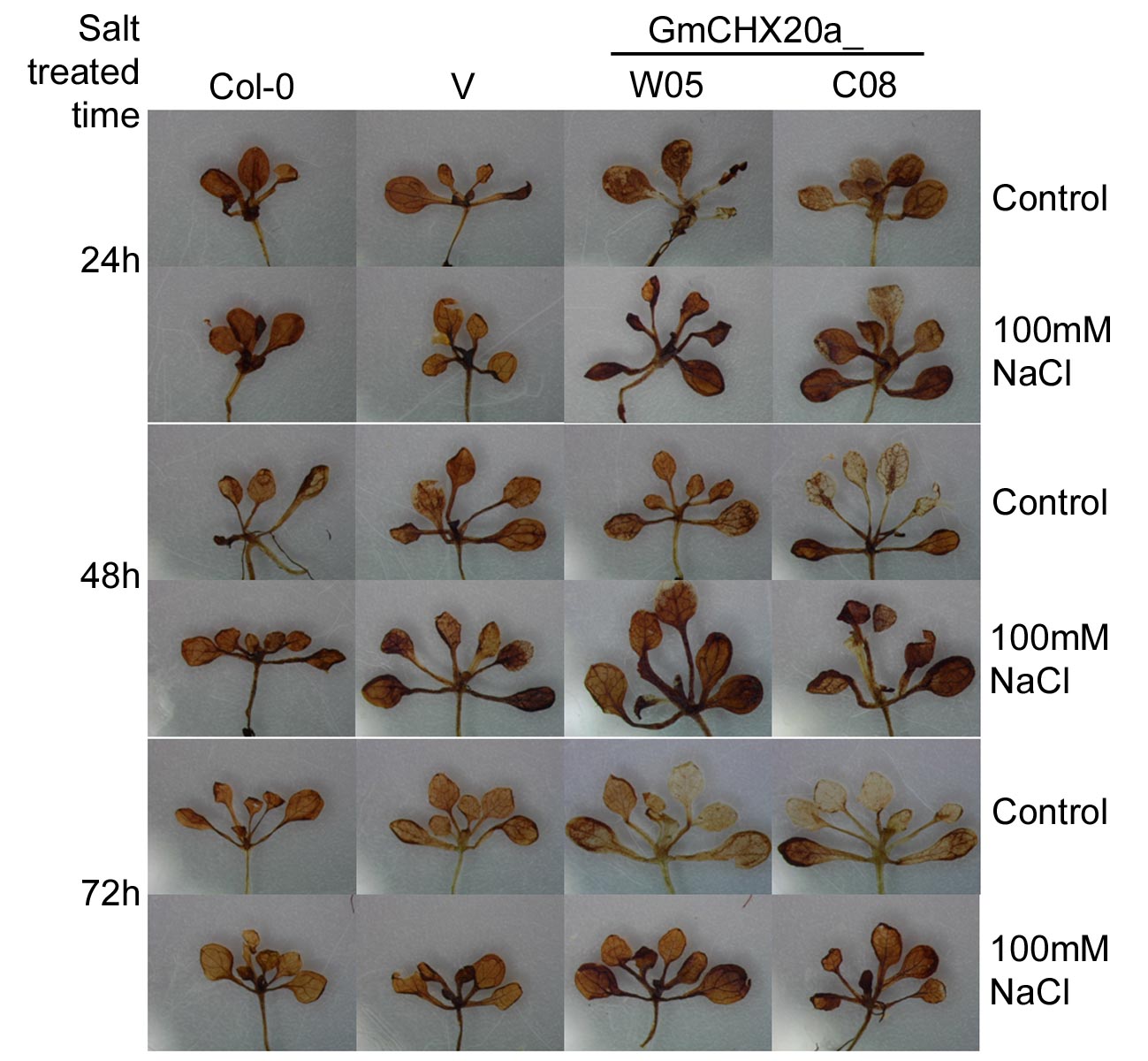

Supplement: Supplementary file 1 [file ijms-24-16560-s001.zip › supplementarypv1/suplementary materials20231106/Figure S3-CHXDAB.jpg]
